# Podoplanin mediates ECM degradation by squamous carcinoma cells through control of invadopodia stability

**DOI:** 10.1038/onc.2014.388

**Published:** 2014-12-08

**Authors:** E Martín-Villar, B Borda-d'Agua, P Carrasco-Ramirez, J Renart, M Parsons, M Quintanilla, G E Jones

**Affiliations:** 1 Instituto de Investigaciones Biomédicas ‘Alberto Sols' (CSIC-UAM), Madrid, Spain; 2 Randall Division of Cell & Molecular Biophysics, King's College London, London, UK

## Abstract

Invadopodia are actin-rich cell membrane projections used by invasive cells to penetrate the basement membrane. Control of invadopodia stability is critical for efficient degradation of the extracellular matrix (ECM); however, the underlying molecular mechanisms remain poorly understood. Here, we uncover a new role for podoplanin, a transmembrane glycoprotein closely associated with malignant progression of squamous cell carcinomas (SCCs), in the regulation of invadopodia-mediated matrix degradation. Podoplanin downregulation in SCC cells impairs invadopodia stability, thereby reducing the efficiency of ECM degradation. We report podoplanin as a novel component of invadopodia-associated adhesion rings, where it clusters prior to matrix degradation. Early podoplanin recruitment to invadopodia is dependent on lipid rafts, whereas ezrin/moesin proteins mediate podoplanin ring assembly. Finally, we demonstrate that podoplanin regulates invadopodia maturation by acting upstream of the ROCK-LIMK-Cofilin pathway through the control of RhoC GTPase activity. Thus, podoplanin has a key role in the regulation of invadopodia function in SCC cells, controlling the initial steps of cancer cell invasion.

## Introduction

Approximately 90% of human tumours arise from epithelial tissues, which are separated from the supporting stroma by the basement membrane (BM).^1^ Invasion of cancer cells through this physiological barrier represents a key step during cancer progression, because it is only within the supporting stroma that cancer cells gain access to the vascular and lymphatic systems and spread systemically. Despite current efforts to understand cancer cell invasion, the mechanisms underlying BM transmigration by tumour cells remain elusive. Accumulating evidence demonstrates that cancer cells overcome the BM barrier by forming specialised F-actin-rich projections termed invadopodia, which serve as a localised source of matrix proteases and mediate the degradation of the extracellular matrix (ECM).^1, 2, 3^ A four-step model for invadopodia assembly has been defined revealing that stabilisation and complete maturation of invadopodia are required for efficient ECM degradation.^4, 5, 6^ Although several studies have described their structural components,^7, 8, 9^ there is a great interest in understanding the regulatory events controlling invadopodia stability and maturation.^10, 11, 12^

Squamous cell carcinoma (SCC) is a malignant tumour of stratified epithelia characterised by invasive growth into the connective tissue. Interestingly, primary cancer cells obtained from patients with invasive SCCs spontaneously assemble active invadopodia when cultured in two-dimensional substrates.^13^ Podoplanin is a type I transmembrane mucin widely known as a marker for lymphatic endothelial cells that has a critical role during development of the heart, lungs and lymphatic endothelial system.^14, 15, 16^ The expression of podoplanin is significantly enhanced during tissue-remodelling processes and in several types of human cancer, including SCCs,^17, 18, 19^ where podoplanin upregulation is often found at the leading invasive edge of tumour nests and is generally associated with poor clinical outcome.^20, 21, 22, 23, 24^ *In vitro*, podoplanin expression in tumour cells is linked to enhanced migratory and invasive features.^19, 25^ Among the best defined partners for podoplanin are the C-type adhesion receptor 2 (Clec-2),^25^ the adhesion molecule CD44 and the ERM (Ezrin, Radixin, Moesin) protein family, which link the underlying actin cytoskeleton to podoplanin cytoplasmic (CT) tail. Podoplanin binding to the standard isoform of CD44 in carcinoma cells seems crucial for podoplanin-dependent stimulation of directional migration^26^ as well as for tethering tumour cells to hyaluronan-rich ECM.^27^ In addition, the binding of podoplanin to ERM proteins triggers the activation of the small GTPase RhoA, regulating actin dynamics.^28^ Podoplanin-mediated regulation of the actin cytoskeleton is often associated with the acquisition of a mesenchymal phenotype (epithelial mesenchymal transition; EMT), although podoplanin can also stimulate tumour progression in the absence of a complete EMT.^23^ *In vitro*, podoplanin-mediated invasion depends on the activity of matrix metalloproteinases;^23^ however, the underlying molecular mechanisms remain poorly understood.

In this study, we sought to investigate whether podoplanin pro-invasive properties could be related to invadopodia activity in SCC cells. We describe podoplanin as a novel component of invadopodia adhesion rings, where it controls invadopodia stability and promotes efficient degradation of the ECM. We also characterise the molecular mechanisms underlying this novel function of podoplanin. These results demonstrate a direct involvement of podoplanin in ECM-remodelling processes.

## Results

### Podoplanin defines adhesion rings at invadopodia of SCC cells

**Figure 1:**
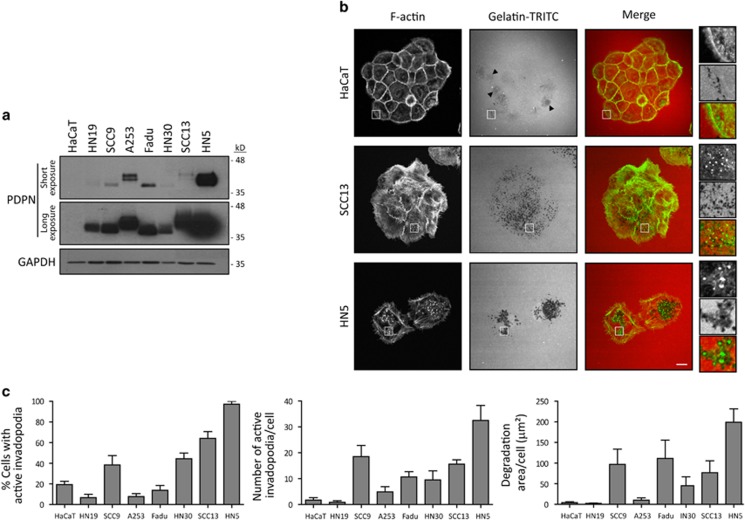
Correlation between endogenous podoplanin expression and invadopodia formation in human SCC cell lines. (**a**) Endogenous podoplanin expression in subconfluent SCC cell cultures by western blot. Two different exposure times are shown (upper panel 15 s; lower panel 40 s). The molecular mass variability observed between the different cell lines arises from the presence of O-linked carbohydrates on its extracellular domain as previously reported.^21, 26, 54^ (**b**) Determination of active invadopodia formation in SCC cells by gelatin-degradation assays. The different SCC cell lines were cultured on glass coverslips covered with crosslinked TRITC-gelatin for 18 h. Representative confocal images of SCC cells are shown. Insets show zoomed areas of individual invadopodia. Bar=10 μm. (**c**) Quantification of active invadopodia formation in SCC cells (left and middle) and degradation area per cell (right) by gelatin-degradation assay. The results shown are the means±s.e.m. of *n⩾*100 cells (left graph) or *n⩾*10 cells-100 invadopodia (middle and right graphs) for each condition over three independent experiments.

We have previously shown that podoplanin is absent in non-transformed human and mouse keratinocytes *in vitro*.^17, 21^ As podoplanin is commonly upregulated in human SCCs, we analysed its expression in a panel of human SCC cell lines. Overall, SCC cells showed high levels of podoplanin compared with non-transformed HaCaT keratinocytes (Figure 1a). To determine whether podoplanin pro-invasive properties could be related to invadopodia activity in SCC cells, we first quantified the ability of the different cell lines to assemble invadopodia when plated on TRITC-gelatin. After 18 h, all cell lines showed actin-rich protrusions associated with dot-like areas of matrix degradation, characteristic of active invadopodia. Although podoplanin protein levels did not directly correlate with the number of cells able to form invadopodia, SCC cell lines exhibiting higher podoplanin protein levels (for example, HN5) formed robust invadopodia associated with areas of focalised ECM degradation, whereas those showing lower podoplanin expression (for example, SCC13) often presented smaller invadopodia associated with more superficial and diffuse areas of matrix degradation (Figures 1b and c).

**Figure 2:**
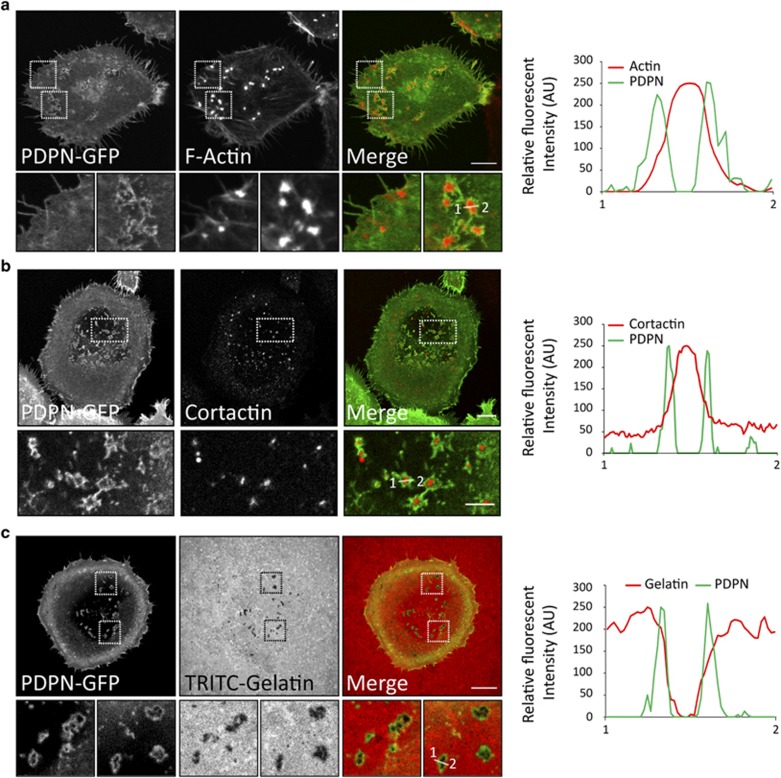
Podoplanin defines adhesion rings at invadopodia of SCC cells. Confocal images showing specific localisation of podoplanin fused to GFP (PDPN-GFP) at invadopodia in HN5 cells cultured on glass coverslips covered with crosslinked gelatin (**a, b**) or TRITC-gelatin (**c**). Note that podoplanin is not present in all invadopodia (**a**; left lower panels) but clusters to active invadopodia forming a ring structure around the actin or cortactin core (**a, b**; lower panels and **c**). Graphs indicate fluorescent intensity (in arbitrary units) of PDPN-GFP with respect to F-actin or cortactin over the indicated line scan. Data shown are representative from six invadopodia analysed per condition from two independent experiments. Bars=10 μm.

We next investigated whether podoplanin is a component of invadopodia in SCC cells. The presence of both endogenous and GFP-tagged podoplanin (PDPN-GFP) at invadopodia was confirmed by immunofluorescence co-staining with the invadopodium markers actin and cortactin, and colocalisation with areas of matrix degradation. Confocal microscopy analysis revealed that podoplanin was mostly absent from the invadopodia actin core but accumulated to podosome-like adhesion rings of actively degrading invadopodia in HN5 and SCC13 cells (Figure 2, Supplementary Figure S1A and S2A). The presence of adhesion rings in these cells was confirmed by co-staining of vinculin, a component of invadopodia adhesion rings^7^ and the adhesion molecule talin (Supplementary Figure S1B and C). The localisation of podoplanin at invadopodium rings was also confirmed in MDA-MB-231 breast adenocarcinoma cells upon podoplanin ectopic expression (Supplementary Figure S3A and C). These results demonstrate that podoplanin is a novel component of invadopodium adhesion rings and that this specific localisation is not cell-type dependent.

### Genetic manipulation of podoplanin reveals a role in invadopodia-mediated matrix degradation

**Figure 3:**
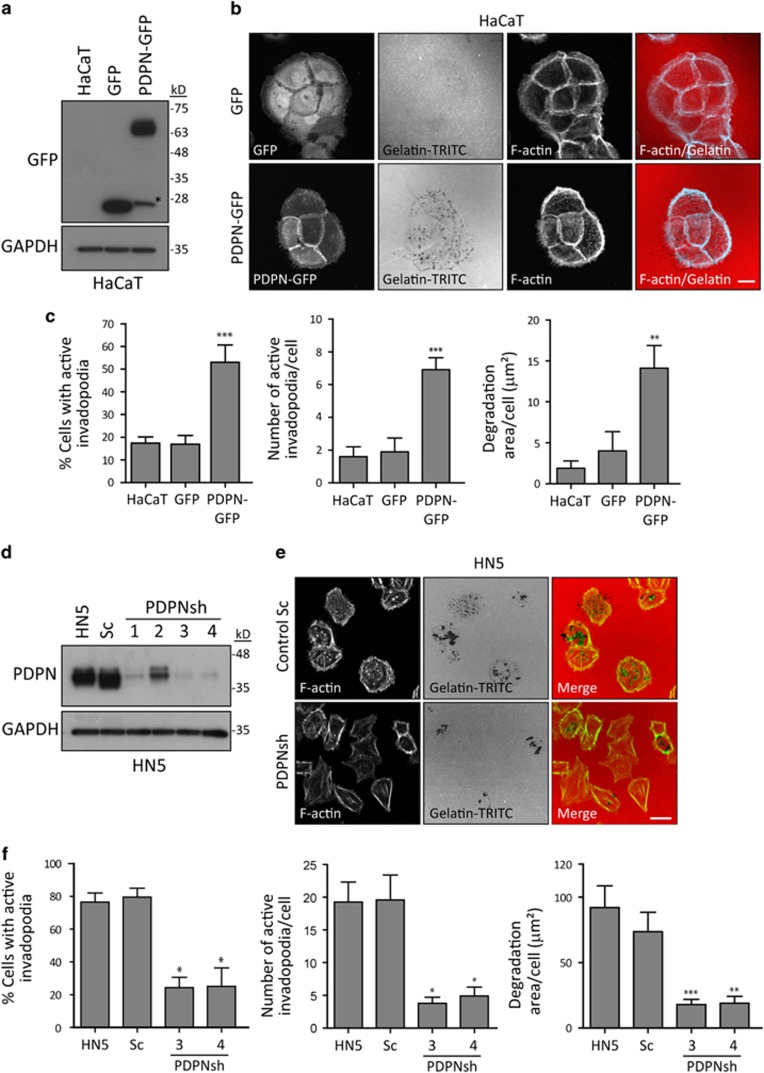
Podoplanin is required for efficient invadopodium-associated matrix degradation in SCC cells. (**a**) Western blot of whole-cell lysates from HaCaT cells stably transfected with GFP (HaCaT-GFP) or GFP-tagged podoplanin (HaCaT PDPN-GFP) blotted for GFP and GAPDH (as loading control). Podoplanin undergoes proteolytic cleavage leading to the liberation of its intracellular domain into the cytosol (asterisk).^55^ (**b**) Gelatin-degradation assay using HaCaT cell transfectants (8h). Bar=25 μm. (**c**) Quantification of the gelatin-degradation assay depicted in **b**. Graphs represent the percentage of cells containing actin-rich protrusions that degrade the gelatin (left), number of active invadopodia per cell (middle) and degradation area per cell (right). Results shown are the means±s.e.m. of *n⩾*100 cells (left graph) or *n⩾*10 cells-100 invadopodia (middle and right graphs) over three independent experiments. (**d**) Western blot showing podoplanin downregulation in HN5 cells. (**e**) Gelatin-degradation assay using podoplanin-depleted HN5 cells (6 h). Podoplanin-specific silencing by shRNAs decreases active invadopodia formation in HN5 cells. Bar=25 μm. (**f**) Quantification of the gelatin degradation assay represented in **e**. Results shown are the means±s.e.m. of *n⩾*100 cells (left graph) or *n⩾*10 cells-100 invadopodia (middle and right graphs) for each condition over three independent experiments. ***P<0.0001; **P<0.001; *P<0.01.

The specific localisation of podoplanin to invadopodia suggested that this glycoprotein may have a role in the formation and/or function of invadopodia. To investigate this, we analysed the effect of podoplanin ectopic expression on invadopodia-mediated ECM degradation using HaCaT cells. Control GFP or HaCaT cells showed few and very small invadopodia sometimes only visible by colocalisation with discrete areas of degradative dots; however, PDPN-GFP expressing cells exhibited more robust invadopodia, which was associated with a significant increase in the number of active invadopodia and the extent of matrix degradation (Figures 3a–c and Supplementary Figure S4). Likewise, ectopic expression of podoplanin in MDA-MB-231 cells, which spontaneously form invadopodia,^4^ led to a further increase in the number of active invadopodia (Supplementary Figure S3E and F). To verify these results, we analysed whether the downregulation of endogenous podoplanin affected the function of invadopodia in SCC cells. We selected the cell lines HN5 and SCC13 because they constitutively assemble functional invadopodia and express very high or moderate levels of podoplanin, respectively (Figure 1). Podoplanin was almost completely depleted (∼90%) by using the specific shRNAs 3 and 4 in both cell lines (Figure 3d and Supplementary Figure S2B). Consistent with the previous observations, invadopodia-mediated ECM degradation was significantly reduced in HN5 PDPNsh cells in comparison with parental or control Sc cells, in which the majority of cells formed proteolytically active invadopodia (Figures 3e and f). A significant reduction in the number of active invadopodia and associated ECM degradation was also observed after podoplanin silencing in SCC13 cells (Supplementary Figure S2C and D). Conversely, podoplanin overexpression in SCC13 cells significantly enhanced invadopodia-mediated ECM degradation (Supplementary Figure S2C, E). Together, these results strongly indicate that podoplanin enhances invadopodia-mediated matrix degradation.

### Podoplanin promotes invadopodia stabilisation leading to efficient ECM degradation

**Figure 4:**
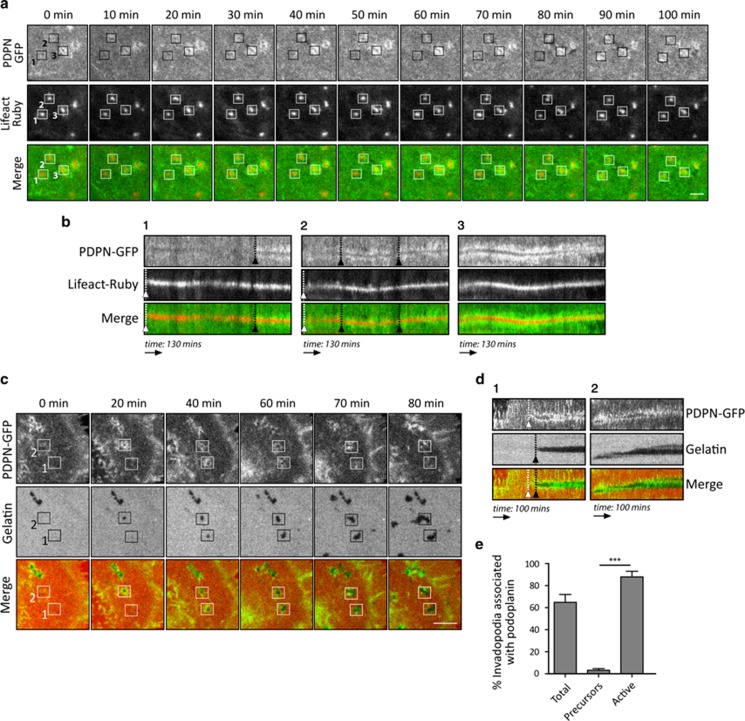
Dynamics of podoplanin during invadopodia assembly and matrix degradation. (**a**) Podoplanin rings form following actin polymerisation. Live-cell imaging of HN5 cells expressing PDPN-GFP and Lifeact-Ruby cultured on 0.5% glutaraldehyde-crosslinked gelatin. Pictures represent zoomed areas of invadopodia dynamics over time. (**b**) Panels 1, 2, 3 are kymographs showing the lifetime of representative invadopodia from time-lapse imaging in **a** (boxes). White arrowheads indicate invadopodium formation time, and black arrowheads indicate the time of podoplanin ring assembly. (**c**) Podoplanin ring assembly precedes matrix degradation. Time-lapse confocal microscopy of HN5 cells expressing PDPN-GFP cultured on 0.5% glutaraldehyde-crosslinked TRITC-gelatin. (**d**) Panels 1 and 2 show Kymographs representing a subset of invadopodia from time-lapse in **c** (boxes). White arrowheads indicate the time of podoplanin ring assembly, whereas black arrowheads indicate the time of gelatin degradation appearance. (**e**) Graph represents the percentage of podoplanin-ringed invadopodia in the different populations of invadopodia. Invadopodia were classified into two main populations: precursors (newly formed invadopodia with no ability to degrade the matrix) and active (invadopodia actively degrading the matrix). Results shown are the means±s.e.m. of *n⩾*70 invadopodia (five cells) for each condition over two independent experiments. ***P<0.0001. Animate sequences of these data are shown in Supplementary Movies S1 and S2. Bars=5 μm.

Invadopodia initially form as non-proteolytic precursor structures, which polymerise actin and recruit matrix metalloproteinases to develop into fully functional, mature invadopodia.^4, 6^ To understand how podoplanin affects invadopodia function, we analysed the real-time dynamics of podoplanin rings forming at invadopodia. HN5 cells stably expressing PDPN-GFP were transiently transfected with Lifeact-Ruby (to visualise F-actin) and plated onto unlabelled gelatin (Figure 4a and Supplementary Movie S1). In agreement with previous reports,^7, 29^ we frequently observed oscillations of actin intensity at invadopodium puncta. These actin oscillations were also accompanied by oscillations of podoplanin rings that assembled only after actin puncta formation and persisted around the actin core (Figure 4b). To characterise the relationship between podoplanin dynamics and ECM degradation, HN5 PDPN-GFP cells were plated on thin TRITC-labelled gelatin (Figure 4c and Supplementary Movie S2). Live-cell imaging revealed that podoplanin rings were assembled immediately before ECM degradation initiated and the majority persisted during active matrix degradation (Figure 4d). The observation that podoplanin ring assembly precedes ECM degradation but not actin polymerisation strongly indicates that podoplanin does not trigger initiation of invadopodia but rather may enhance invadopodia stability/persistence. Thus, podoplanin was mainly found in active invadopodia but not in invadopodia precursors (Figure 4e).

**Figure 5:**
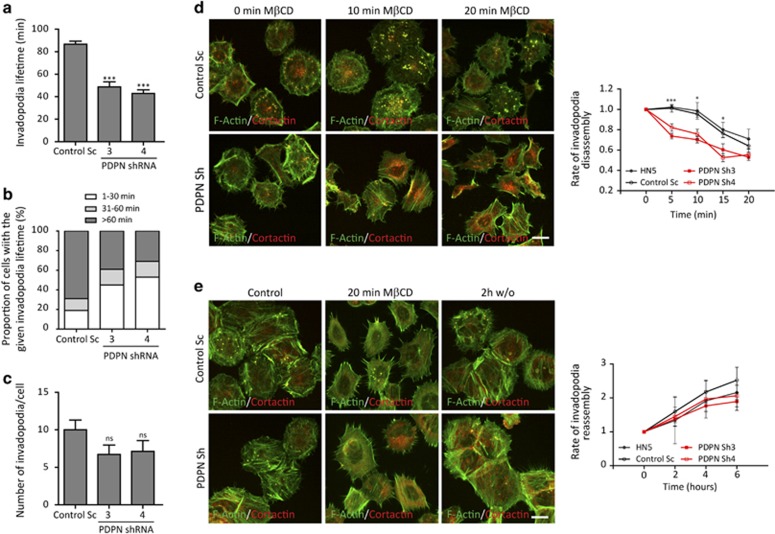
Podoplanin mediates invadopodia stabilisation. (**a**–**c**) HN5 control (Sc) and podoplanin-depleted (PDPN sh3 and sh4) cells expressing Lifeact-GFP were cultured on 0.5% glutaraldehyde-crosslinked gelatin for 3 h. Live-cell imaging was performed and analysed to calculate invadopodia lifetime (**a**, **b**) and total number of invadopodia per cell (**c**). *N⩾*90 invadopodia per condition from 10 cells over three independent experiments. Error bars represent s.e.m. An animate sequence of these data is shown in Supplementary Movie S3. (**d**) Invadopodia disassembly rates in control and podoplanin-depleted cells. Cells were grown on crosslinked unlabelled gelatin overnight. Invadopodia disassembly was induced by treatment with 10mM methil-β-cyclodextrin (MβCD) for 20 min. Cells were fixed at the indicated time points, double stained for actin and cortactin and the number of cells with invadopodia was determined. (**e**) Invadopodia reassembly rates were determined after washout of MβCD and incubation with normal medium. Rates of invadopodia disassembly and reassembly were calculated as the percentage of cells forming invadopodia on each time point. Values were normalised to *t*=0 min (disassembly) or to *t*=20 min after MβCD treatment (reassembly). The results shown are the means±s.e.m. of *n⩾*200 cells for each condition and time point over three independent experiments. Bars=20 μm. ***P<0.0005; * *P*<0.05; NS=not significant.

To test our hypothesis, we investigated the impact of podoplanin expression on invadopodia lifetime. Control and podoplanin-depleted HN5 cells expressing Lifeact-GFP were cultured on unlabelled crosslinked gelatin, and the dynamics of invadopodia analysed by time-lapse confocal microscopy (Figures 5a and c and Supplementary Movie S3). Approximately 70% of invadopodia in control cells were long-lived and stable, often persisting in a single location for time periods >1 h. However, podoplanin-silenced cells showed an approximately twofold reduction in invadopodia lifetime compared with control cells. While only 19% of invadopodia in control Sc cells showed lifetimes <30 min, the population of short-lived invadopodia in podoplanin-depleted cells reached ∼50% (Figure 5b). A trend towards a reduction in the number of invadopodia per cell was also observed in podoplanin-deficient cells, although these differences were not statistically significant (Figure 5c).

To confirm whether podoplanin has a general role in regulating invadopodia stability, we compared the rates of invadopodia disassembly of control and podoplanin-depleted cells. Invadopodia disassembly was induced upon treatment with methyl-β-cyclodextrin (MβCD), which disassembles lipid rafts, inhibiting invadopodia formation and function.^30, 31^ Endorsing our hypothesis, podoplanin-depleted cells disassembled invadopodia faster during the first 5–10 min of treatment, compared to control cells (Figure 5d). Finally, to determine whether podoplanin affects the early steps of invadopodia formation, we took advantage of the reversible effect of MβCD, which allows synchronous invadopodia reassembly upon washout. As shown in Figure 5e, rates of invadopodia reassembly in control and podoplanin-depleted cells were nearly identical, suggesting that initial invadopodia assembly occurs in a podoplanin-independent manner. These results were also confirmed after synchronous induction of invadopodia reassembly by overnight serum starvation and subsequent stimulation with fetal bovine serum (Supplementary Figure S5). Together, these data demonstrate that podoplanin is dispensable for the initial steps of invadopodium precursor formation but is required for the subsequent stabilisation and maturation into fully functional invadopodia.

### Podoplanin linkage to the actin cytoskeleton and lipid rafts-association are necessary for efficient invadopodia-mediated matrix degradation

**Figure 6:**
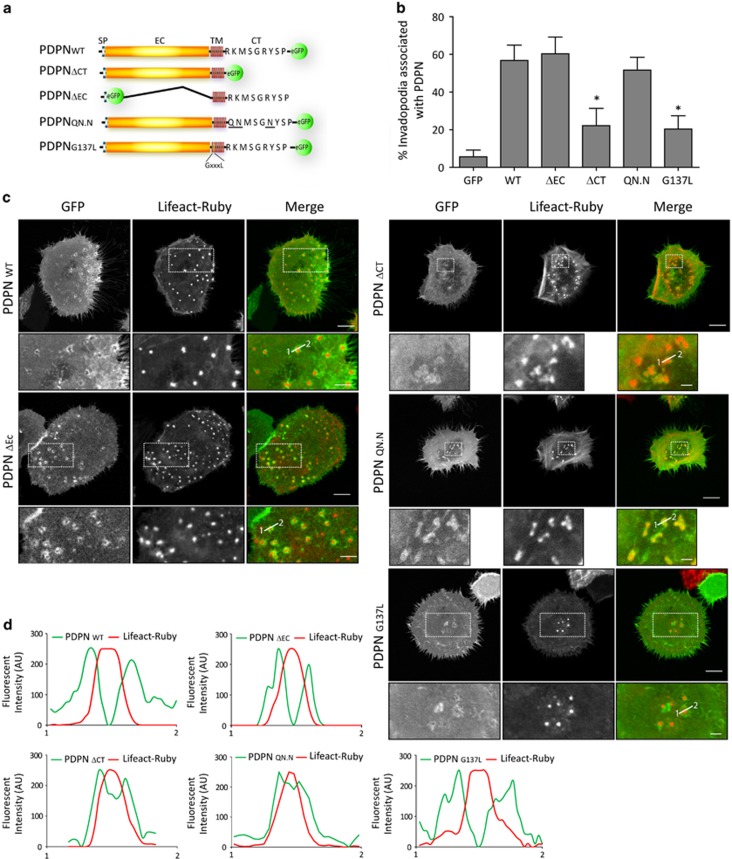
Localisation of wild-type and mutant podoplanin proteins at invadopodia of HN5 cells. (**a**) Schematic representation of podoplanin fusion constructs used for rescue experiments. SP, signal peptide; EC, ectodomain; TM, transmembrane domain; CT, cytoplasmic tail. Basic amino acids (bold) within the CT tail (RK…R) were substituted by uncharged polar residues (QN…*N*; bold and underlined) disrupting the binding site for ERM proteins. The GXXXL motif within the TM domain was disrupted by substitution of the G in the position 137 by L. (**b**) Graph represents the percentage of invadopodia showing recruitment of the indicated podoplanin constructs. The results shown are the means±s.e.m. of *n⩾*250 invadopodia from two independent experiments. *P<0.05. (**c**) Confocal images of HN5 cells expressing the indicated podoplanin proteins. Bars=16 μm (upper panels) and 8 μm (lower panels). (**d**) Graphs indicate fluorescent intensity (in arbitrary units) of each podoplanin mutant construct with respect to F-actin over the indicated line scan. Data shown are representative from 5–8 invadopodia analysed per condition from three independent experiments.

Next, we sought to determine the specific contribution of podoplanin structural domains to its localisation and associated invadopodia stability using several podoplanin mutant constructs previously characterised.^26, 32^ HN5 cells expressing Lifeact-Ruby were transiently transfected with wild-type podoplanin or podoplanin mutant constructs lacking the CT tail (PDPN_ΔCT_), the extracellular domain (PDPN_ΔEC_) or the ERM-binding motif (PDPN_QN.N_). In addition, a podoplanin mutant that prevents podoplanin self-assembly and association to lipid rafts (PDPN_G137L_) was also used (Figure 6a). Confocal microscopy analysis revealed that the recruitment of PDPN_ΔCT_ and PDPN_G137L_ mutants to invadopodia was markedly reduced and could only be observed in few cells, where their assembly into adhesion rings was disrupted (Figure 6b). In these cells, PDPN_ΔCT_ mutant showed partial colocalisation with the actin core of invadopodia, whereas PDPN_G137L_ mutant accumulated diffusely to invadopodia and assembled into wider rings. Interestingly, although PDPN_QN.N_ mutant was recruited to invadopodia, it failed to assemble into rings, and instead colocalised with the actin core, similar to PDPN_ΔCT_. Deletion of the podoplanin ectodomain did not change recruitment or localisation to invadopodia (Figures 6c and d).

**Figure 7:**
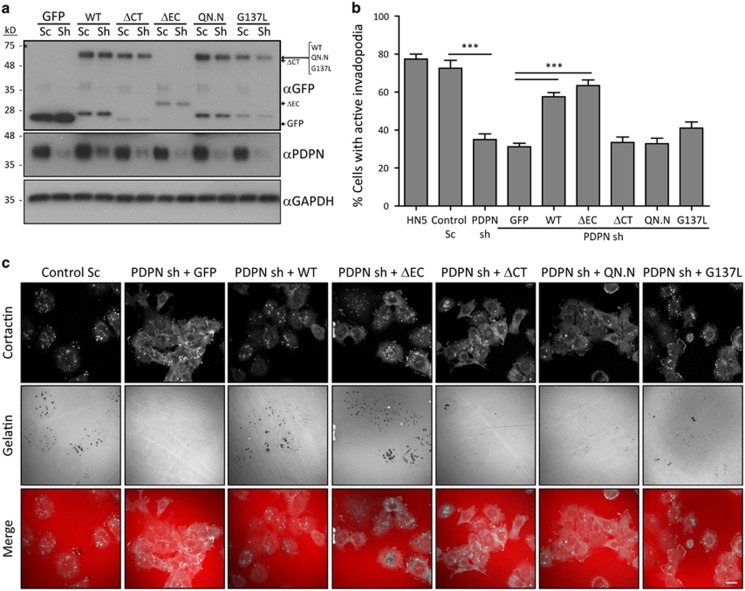
Podoplanin linkage to the cortical actin cytoskeleton and its association to lipid rafts are required for invadopodia-mediated ECM degradation. (**a**) Western blot showing the re-expression of WT podoplanin and mutant constructs into podoplanin-depleted HN5 cells. (**b**, **c**) The ability of WT podoplanin or the indicated mutant constructs to rescue the invadopodia defects induced by depletion of podoplanin was evaluated by gelatin-degradation assays (6 h). Bar graph represents the percentage of cells forming active invadopodia on each condition. (**b**) Representative images of the gelatin-degradation assay depicted in **b** are shown in **c**. Bars=20 μm. The results shown are the means±s.e.m. of *n⩾*100 cells for each condition over three independent experiments. ***P<0.0005.

To determine whether these podoplanin mutants also altered invadopodia-mediated ECM degradation, wild-type podoplanin and mutant constructs were stably expressed into podoplanin-silenced cells (Figure 7a). Cells re-expressing wild-type podoplanin and PDPN_ΔEC_ mutants restored invadopodia functionality in podoplanin-depleted cells (Figures 7b and c), confirming the essential role of podoplanin in invadopodia-mediated ECM degradation. However, PDPN_ΔCT_, PDPN_QN.N_ and PDPN_G137L_ mutants failed to rescue the defects induced by podoplanin knockdown. Altogether, these results reveal that the transmembrane and cytoplasmic domains are the essential motifs for podoplanin recruitment to invadopodia and ECM degradation, pointing to the importance of podoplanin linkage to the actin cytoskeleton, and podoplanin association to lipid rafts for the function of invadopodia.

### ERM proteins mediate podoplanin ring assembly enhancing invadopodia-mediated degradation

**Figure 8:**
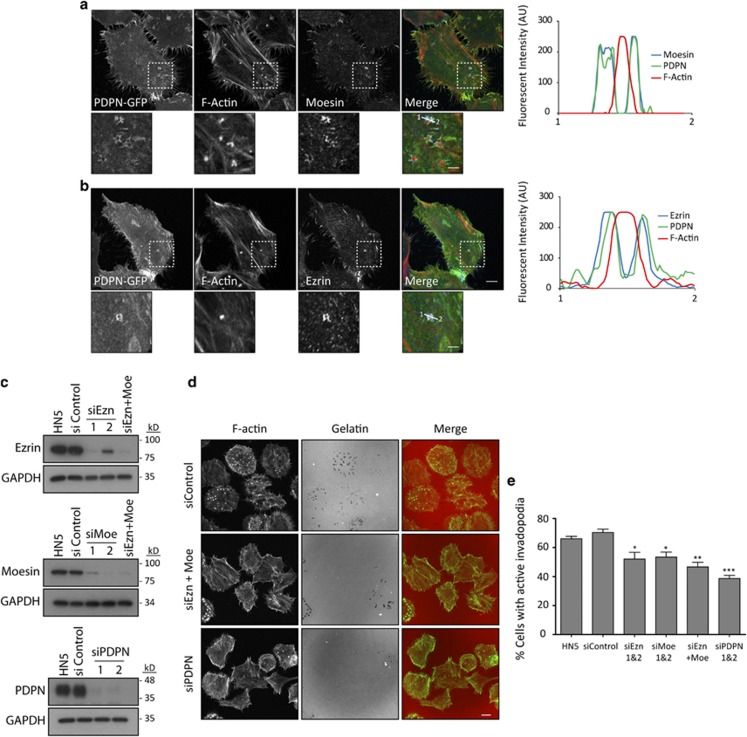
ERM proteins localise at invadopodia adhesion rings and mediate podoplanin ring assembly. (**a, b**) Specific localisation of moesin (**a**) and ezrin (**b**) at invadopodia of HN5 cells cultured on crosslinked gelatin. Note that ezrin and moesin cluster to invadopodia adhesion rings where they colocalise with podoplanin. Graphs indicate fluorescent intensity (in arbitrary units) of each marker over the indicated line scan. Data shown are representative from five invadopodia analysed per condition from three independent experiments. Bars=10 μm (upper panels) and 5 μm (lower panels). (**c**) Western blot analysis of ezrin, moesin and podoplanin expression in HN5 cells upon specific siRNA treatment. (**d**) Gelatin-degradation assay (6 h) of HN5 cells treated with ERM and podoplanin siRNAs. Bars=20 μm. (**e**) Quantification of the gelatin-degradation assay depicted in **d**. The results shown are the means±s.e.m. of *n⩾*100 cells for each condition over three independent experiments. ***P<0.0005; **P<0.005; *P<0.05.

The observed effects of PDPN_QN.N_ mutant suggested that the spatial restriction of podoplanin localisation and function into the adhesion ring of invadopodia are mediated by ERM proteins. To investigate this further, we first analysed whether ezrin and moesin localised to invadopodia of HN5 PDPN-GFP cells. Confocal analysis confirmed that the localisation of both proteins was restricted to invadopodia adhesion rings, where they colocalised with podoplanin (Figures 8a and b). Ezrin and moesin were not present in all invadopodia, similar to that seen with podoplanin. To determine whether ezrin and/or moesin are essential components for invadopodia function in HN5 cells, we used siRNA to deplete these proteins (Figure 8c). As evidenced by gelatin-degradation assays, silencing ezrin or moesin led to a significant reduction in functional invadopodia assembly, which was further decreased by the combined depletion of both molecules (Figures 8d and e). Furthermore, podoplanin localisation in the residual invadopodia of ezrin/moesin-depleted cells was not restricted to the adhesion rings, but instead showed partial colocalisation with the actin core, similar to PDPN_ΔCT_ and PPDPN_QN.N_ mutants (Supplementary Figure S6A). On the other hand, we did not observe recruitment of ERM proteins to the residual invadopodia of podoplanin-depleted cells (Supplementary Figure S6B), suggesting that recruitment of ERM proteins to invadopodia is enhanced by podoplanin. Together, these results indicate that ERM proteins act as linkers between podoplanin and the actin cytoskeleton around the actin core stabilising podoplanin rings.

### Podoplanin regulates cofilin activity through the RhoC-ROCK-LIMK pathway to promote invadopodia-mediated matrix degradation

**Figure 9:**
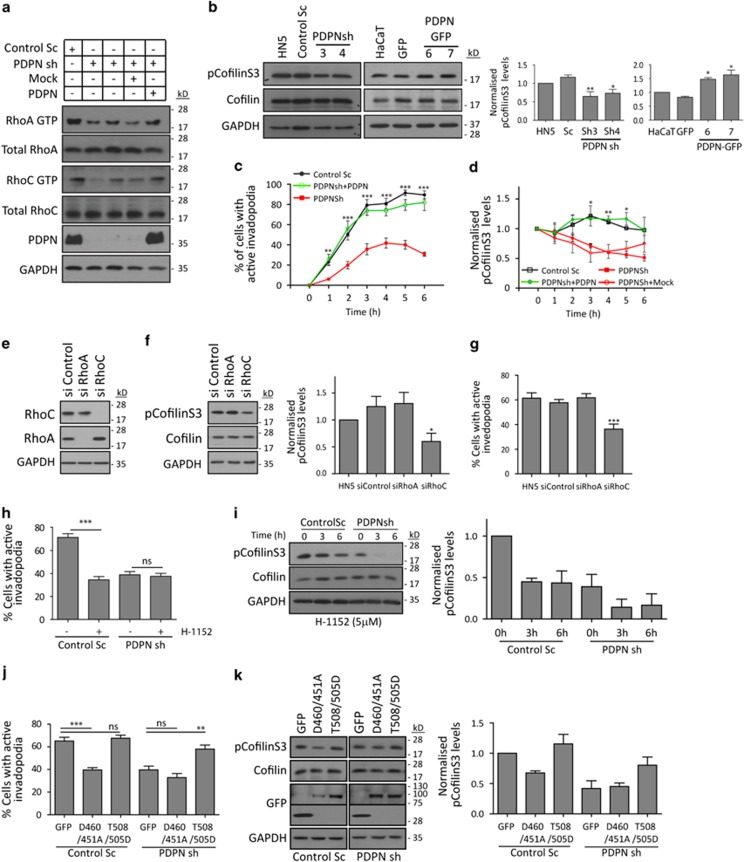
Podoplanin regulates cofilin phosphorylation at Ser3 through the RhoC/ROCK/LIMK pathway. (**a**) Determination of active RhoA and RhoC GTPase levels in control and podoplanin knockdown cells by GTP affinity pull-down assays (see also Supplementary Figure S7B). (**b**) The status of cofilin phosphorylation at Ser3 (pCofilinS3) was evaluated by western blot in podoplanin knockdown HN5 cells and HaCaT cells expressing PDPN-GFP. Quantification of pCofilinS3 levels (right panels) was performed relative to total cofilin levels and GAPDH loading control by densitometric analysis. Values were normalised to HN5 or HaCaT cells to which an arbitrary value of 1 was given. (**c**,**d**) Determination of pCofilinS3 levels during the stages of invadopodia formation in control and podoplanin-depleted HN5 cells. Invadopodia formation in HN5 cell transfectants was synchronised in order to evaluate invadopodia activity and the levels of pCofilinS3 during the stages of invadopodia formation (see Material and Methods section). Invadopodia activity was analysed by gelatin-degradation assay (**c**), and pCofilinS3 changes were monitored by westen blot and quantified by densitometric analysis (**d** and Supplementary Figure S7D). pCofilinS3 levels were normalised to total cofilin levels and GAPDH-loading control. Graphs represent means±s.e.m. of two (**c**) or three (**d**) independent experiments. (**e**) Western blot analysis of RhoC and RhoA expression in HN5 cells upon specific siRNA treatment (see also Supplementary Figure S7C). (**f**) Analysis of pCofilinS3 levels in RhoA- and RhoC-depleted cells. (**g**) Effects of RhoA and RhoC knockdown in invadopodia-mediated degradation of HN5 cells (see also Supplementary Figure S7C). (**h**) Effects of ROCK inhibitor H-1152 in invadopodia-mediated degradation of control Sc and podoplanin-depleted HN5 cells. (**i**) Western blot analysis pCofilinS3 levels after H-1152 treatment. Quantification of pCofilinS3 levels is shown in the right panel. (**j**) Invadopodia-mediated gelatin degradation upon expression of the indicated LIMK1/2 mutant constructs in control Sc and podoplanin-depleted HN5 cells. (**k**) Western blot analysis of pCofilinS3 and LIMK1/2 mutant expression (GFP). Quantification of pCofilinS3 levels is shown in the right panel. All Graphs represent means±s.e.m. of three or four (**d** and **e**) independent experiments. *P<0.01; **P<0.001; ***P<0.0001; NS=not significant.

Some of the major regulatory signals for invadopodia assembly and maturation originate from Rho GTPases.^8^ Cdc42 has been implicated in the actin nucleation necessary for invadopodia formation,^33^ whereas RhoA and RhoC are essential for the control of invadopodia-mediated degradation.^10, 34^ To ascertain whether podoplanin knockdown in HN5 cells disrupts Rho GTPase activity, we used pull-down assays to evaluate the activation status of RhoA, Rac1, Cdc42 and RhoC. In agreement with previous observations,^28^ RhoA but not Rac1 or Cdc42 activity was significantly reduced in podoplanin-depleted cells with respect to control cells (Figure 9a and Supplementary Figure S7A and B). Interestingly, RhoC activity was also reduced in podoplanin-depleted cells, suggesting that not only RhoA but also RhoC could mediate the function of podoplanin in invadopodia. The Rho/ROCK (Rho-associated protein kinase) pathway has been involved in invadopodia maturation through control of cofilin phosphorylation.^10^ Podoplanin knockdown cells showed a very small but consistent reduction of cofilin phosphorylation at Ser3 (pCofilinS3; Figure 9b). Conversely, pCofilinS3 was slightly increased upon ectopic expression of podoplanin in HaCaT cells (Figure 9b). To further define the kinetics of pCofilinS3 during invadopodia assembly, control and podoplanin-silenced HN5 cells were serum-starved overnight and stimulated with fetal bovine serum to synchronously induce invadopodia formation and associated matrix degradation (Figure 9c). Formation of non-functional immature invadopodia was observed during the first 30 min after stimulation in control cells. Matrix degradation initiated after 1h, and ∼90% of control cells showed fully mature invadopodia after 3-4 h, by which point the cells were no longer synchronised. pCofilinS3 levels were slightly decreased in control cells at 0–1h, correlating with initiation of invadopodia formation and actin polymerisation (Figure 9d and Supplementary Figure S7D). After 1 h, pCofilinS3 levels were increased accordingly with the stabilisation and maturation steps (2–4 h). Podoplanin Knockdown led to a global and steady decrease in pCofilinS3 compared with control cells. Restoration of podoplanin expression in PDPNsh cells (Figure 9a) rescued the observed defects in matrix degradation and pCofilinS3.

As both RhoA and RhoC can regulate cofilin phosphorylation through the activation of ROCK,^35^ we sought to determine which GTPase isoform is responsible for the regulation pCofilinS3 levels in HN5 cells using specific siRNAs. RhoC Knockdown resulted in a significant upregulation of RhoA levels, whereas RhoC expression was unaltered after RhoA silencing (Figure 9e and Supplementary Figure S7C). Despite this observed RhoA upregulation, RhoC-depleted cells showed a significant reduction in functional invadopodia assembly, which was also associated to decreased pCofilinS3 levels (Figures 9e and f). These results strongly indicate that RhoC, but not RhoA, is the GTPase isoform controlling the phosphorylation status of cofilin and invadopodia function in HN5 cells. Thus, RhoA silencing did not affect invadopodia activity or pCofilinS3 levels (Figures 9f and g). However, we also observed a significant upregulation of Rac1 and Cdc42 after RhoA depletion (Supplementary Figure S7F), probably masking the specific RhoA effects. These results suggest that podoplanin regulates cofilin activity though the activation of RhoC but not RhoA.

Finally, we investigated whether podoplanin-dependent cofilin phosphorylation in HN5 cells is regulated by ROCK and LIMK (LIM-domain kinase). Treatment of control cells with a specific inhibitor of ROCK (H-1152) reduced pCofilinS3 and invadopodia-mediated matrix degradation to levels seen with podoplanin knockdown (Figures 9h and i). However, matrix degradation was not further decreased by treatment with H-1152 in podoplanin-depleted cells despite the drug inducing a further reduction in pCofilinS3 levels. To test the involvement of LIMK in podoplanin-mediated stabilisation of invadopodia, we evaluated the effects of expression of LIMK1/2 mutants on invadopodia function in HN5 cell transfectants. In agreement with previous reports,^36^ expression of the catalytically inactive LIMK1(D460A)/LIMK2(D451A) mutants significantly reduced the ability of control cells to degrade the matrix, whereas no significant effect was observed upon expression in podoplanin knockdown cells (Figures 9j and k). Conversely, expression of the phosphomimetic LIMK1(T508D)/LIMK2(T505D) mutants in control cells had no significant effect on pCofilinS3 and invadopodia activity, whereas it rescued ECM degradation and cofilin activity in podoplanin-depleted cells. Altogether, these results demonstrate that podoplanin modulates the activity of RhoC in order to regulate the ROCK-LIMK pathway that finally leads to controlled regulation of cofilin activity by Ser3 phosphorylation and the subsequent invadopodia stability/maturation.

## Discussion

ECM degradation is critical for the invasion of carcinomas surrounded by dense crosslinked BMs.^37^ Growing evidence suggest that carcinoma cells do not entirely digest the BM, but rather make small perforations that are the result of efficient invadopodial activity.^38, 39^ Podoplanin is highly upregulated at the early stages of SCC progression^17, 20, 22, 24^ and in physiopathological situations involving tissue remodelling.^17, 18^ In all these situations, podoplanin expression is induced in the basal layer of keratinocytes that are in close contact with the BM. Although the mechanisms controlling this upregulation are completely unknown, podoplanin expression can be modulated by a number of factors, including those with the potential to induce invadopodia formation/function such as EGF and TGFβ.^18, 23, 40, 41^ Our present study suggests that podoplanin mediates ECM remodelling in all these contexts by controlling the stability of invadopodia in keratinocytes, pointing to a direct role for this glycoprotein in cancer cell adhesion and invasion. Our data are consistent with a recent report showing the association of podoplanin with invadopodia, although its specific localisation and functional role in these structures was not addressed.^42^ On the basis of the data presented here, we propose a model in which podoplanin is dispensable for the initial formation of invadopodia but promotes invadopodia stabilisation and maturation, leading to efficient ECM degradation (Supplementary Figure S8). Podoplanin association to lipid rafts is essential for its recruitment to invadopodia, where ERM proteins mediate and stabilise the assembly of podoplanin into adhesion rings. Furthermore, our data demonstrate that podoplanin regulates invadopodia maturation by acting upstream of the ROCK-LIMK-Cofilin pathway through the regulation of RhoC GTPase activity.

It is worth noticing that podoplanin is specifically located at invadopodia but it is not a component of focal adhesions or podosomes of differentiated THP-1 cells (Supplementary Figure S1D-E), pointing to podoplanin as a specific component of invadopodia. We demonstrate for the first time that podoplanin is differentially located at invadopodia adhesion rings, where it exists in close association with ezrin/moesin. Although the presence of invadopodia-associated adhesion structures has been controversial,^8, 43, 44^ increasing evidence indicates that adhesion rings surrounding the actin core of invadopodia are essential for the maturation and control of ECM degradation efficiency.^7, 45, 46^ Invadopodia architecture appears to be different depending on the cell type. Thus, some of the recently reported components of adhesion rings such as β1 integrin,^7^ or talin and ezrin/moesin (this work), have also been found in the invadopodia actin core in different cell types,^45, 47, 48^ which may account for the ECM degradation efficiency. Therefore, it is essential to understand how this differential localisation affects the invasive potential of tumour cells and how the specific segregation of invadopodial components into the adhesion ring/actin core is controlled. Our results showing that ERM proteins mediate podoplanin ring assembly suggest an important function for ERM proteins in the spatial organisation of essential invadopodia components in order to regulate their functionality, a hypothesis that warrants further investigation.

Podoplanin association to lipid rafts is essential for efficient podopolanin recruitment to invadopodia and associated ECM degradation. Invadopodia are lipid rafts-enriched domains whose assembly and function are dependent on these microdomains.^30, 31^ It has been proposed that lipid rafts act as platforms for localising and activating the molecular machinery required for invadopodia formation and function.^49^ Our results support this hypothesis, and are in agreement with our previous observations, evidencing the importance of podoplanin recruitment to lipid microdomains for its functionality.^32^ Interestingly, ezrin has also been shown to facilitate the formation of a lipid raft localised signalling complex promoting NHE1-dependent invadopodia formation and proteolytic activity.^47^

Besides the importance of podoplanin CT motif for lipid raft association,^32^ this domain is essential for podoplanin-induced invadopodia stability through the activation of the RhoC-cofilin pathway. Regulation of cofilin activity is a critical event for the maturation of invadopodia precursors into fully active invadopodia.^50, 51^ Although cofilin activation is required for actin polymerisation after invadopodia precursor formation,^6^ the actin severing activity of cofilin has to be finely regulated in order to prevent from excessive severing and actin depolymerisation, allowing the stabilisation of the invadopodium. Interestingly, in metastatic mammary adenocarcinoma cells RhoC activity is spatially confined to areas surrounding the actin core of invadopodia, which in turn increase pCofilinS3 and therefore inactivation, focusing cofilin activity to the actin core.^10^ Thus, podoplanin might be controlling RhoC activity specifically at adhesion rings, allowing the inactivation of cofilin in this specific compartment of invadopodia.

In summary, the results presented here show the functional role of podoplanin in modulating matrix remodelling through invadopodia. Together with previous data, these results demonstrate the involvement of podoplanin in two key features of aggressive tumours: increased directed cell motility and efficient remodelling of the ECM. Our results also demonstrate that invadopodia adhesion rings regulate the efficiency of ECM degradation, highlighting the interest of molecules that regulate the invadopodium adhesion ring assembly such as ERM proteins. Understanding the key signalling molecules that regulate the crucial stages in the invadopodium life cycle will enable the development of targeted therapies designed at preventing cancer cell invasion.

## Materials and methods

### Cell culture

Characteristics and cell culture conditions of SCC cell lines are described in Supplementary Table S1. All cell lines were short tandem repeat (STR) profiled and their genotypes were compared with that of the database (ATCC STR database or Zhao and coworkers^52^). All cell lines were maintained at 37 °C in a 5% CO_2_ humidified atmosphere. HaCaT PDPN-GFP and HN5 PDPNshRNA cell transfectants have been described before.^21, 26^ For the experiments described below, cells were cultured on 0.5% glutaraldehyde-crosslinked gelatin or TRITC-gelatin where indicated.

### Invadopodia gelatin-degradation assays

The matrix-degradation assay has been performed as described previously.^13^ To assess the ability of cells to form invadopodia and degrade matrix, 3–4 × 10^5^ cells were plated on the coated coverslips in six-well plates containing three coverslips/well and incubated at 37 °C for the indicated time (6–18 h). Cells and images were then processed as indicated below.

### Confocal microscopy and invadopodium analysis

Cells plated on labelled or unlabelled crosslinked gelatin were fixed after the indicated time points in 3.7% formaldehyde and processed as previously described.^26^ Fixed cell images were taken using a NIKON A1R confocal laser-scanning microscope equipped with a Plan Apo VC x60 Oil 1.4NA or Plan Fluor x40 Oil 1.3NA objective lenses or Zeiss LSM510 confocal microscope using a × 63 oil objective. Images were captured using NIS Elements software (Nikon Instruments UK, Surrey, UK) and analysed using FIJI (http://fiji.sc/Fiji). Invadopodia were identified as morphologically characteristic actin-cortactin dots (⩾1 μm) found at the bottom of the cell. Foci of degraded matrix were visible as dark dot-like areas that lack fluorescence and appear as ‘holes' in the bright fluorescent gelatin matrix. For the analysis of invadopodia-mediated ECM degradation, only those invadopodia associated with ECM degradation were considered as active. The number of cells forming active invadopodia was manually counted. A cell containing at least one active invadopodia under the cell body or near the cell edge was counted as a cell-forming active invadopodia. The area of gelatin degradation was measured using an ImageJ plugin previously described.^53^

For podoplanin-actin and podoplanin-gelatin imaging, cells stably expressing PDPN-GFP were transiently transfected with Lifeact-Ruby when indicated. After 24 h, the basal cell surface was imaged every 30 s for 3 h by the NIKON A1 confocal system previously described using the × 60 objective. Image acquisition was performed using NIS Elements software. Playback rates of movies shown are 10 frames/s. Kymography analysis was performed using FIJI by selecting areas covering one single invadopodium.

For live-cell invadopodium analysis, raw intensity data were first corrected for background fading during imaging. The actin-containing invadopodia were thresholded, traced at their largest area and fluorescence intensity was then determined at each time point. With this data invadopodium lifetime and total number of invadopodia/cell were determined as described in the figure legend. These analyses were performed using FIJI software.

### Invadopodia assembly experiments and synchronisation

Control and PDPN knockdown cells were seeded on glass coverslips coated with unlabelled crosslinked gelatin and allowed to spread for 3 h in normal medium. To evaluate the rate of invadopodia disassembly 10 mM MβCD was added to the medium at the times indicated for a maximum of 20 min. Cells were fixed and processed as described above and co-stained with phalloidin and cortactin to visualise invadopodia. For reassembly experiments, cells were treated with 10 mM MβCD for 20 min, washed with normal medium and allowed to resume invadopodia formation at the indicated times for a maximum of 6 h.

For synchronisation experiments, cells were seeded on glass coverslips coated with TRITC-crosslinked gelatin or plastic petri dishes coated with unlabelled crosslinked gelatin and serum-starved overnight in the presence of 50 μM G6001 to prevent invadopodia formation and activity. Invadopodia formation and function was resumed after G6001 washout and addition of fresh medium supplemented with 10% fetal bovine serum at the indicated time points. Under these conditions, HN5 cells form inactive invadopodia during the first 30 min after washout and actively degrading invadopodia after 1 h. Cells were then processed for ECM degradation analysis or western blot as in the supplemental methods.

### Statistics

Three to four independent experiments were carried out for each experimental condition. Data are presented as mean±s.e.m. Significance was determined using one-way analysis of variance followed by Bonferroni's multiple comparison test. Data were considered as significant if *P*<0.05 was reached. Significance values and *N* numbers for each data set are shown in each figure legend. All statistical analyses were performed using GraphPad Prism 5.0 software.
