# Supplementary material for: Podoplanin mediates ECM degradation by squamous carcinoma cells through control of invadopodia stability
Source: Oncogene. 2014 Dec 8;34(34):4531–44. doi: 10.1038/onc.2014.388 (PMC4430312; doi:10.1038/onc.2014.388)
Supplement: Supplementary Information [file onc2014388x1.doc]

**SUPPLEMENTARY INFORMATION**

**SUPPLEMENTAL METHODS**

**Reagents and antibodies**

Antibodies were from the following sources: rat anti-human podoplanin (clone NZ1), used for Western blot analysis, was from Acris Antibodies (Herford, Germany), rabbit anti-human podoplanin (used for immunofluorescence analysis) has been described before (Martin-Villar et al., 2005), GAPDH (clone 6C5), cortactin (4F11), p34-ARC/ARPC2 and MT1-MMP (LEM-2/15.8) were from Millipore (Watford, UK), vinculin (clone h-VIN1) and ezrin (3C12) from Sigma, moesin (Q480), Cdc42, N-WASP total cofilin, pCofilinS3 and RhoC (D40E4) from Cell Signalling Technology (Danvers, MA, USA), RhoA (26C4) and WIP from Santa Cruz Biotechnology (Santa Cruz, CA), mouse anti-GFP from Roche Diagnostics, mouse anti-fascin was from Dako (Cambridge, UK), Rac1 was from BD Biosciences and pCortactinY421 was from Chemicon International. Anti-rabbit and mouse horseradish peroxidase-conjugated secondary antibodies were from Dako, anti-rat and anti-goat from Santa Cruz Biotechnology. Alexa Fluor fluorescent secondary antibodies and Phalloidin were from Invitrogen (Grand Island, NY, USA). Methyl-β-cyclodextrin was purchased from Sigma and used at 10 mM for 20 min prior to washout or fixation. ROCK inhibitor (H-1152) was purchased from Calbiochem and used at 5 μM from 3-6 h before the cells were fixed or lysed. MMP broad spectrum inhibitor G6001 was used at 50 μM and purchased from Millipore. Phorbol 12,13-dibutyrate (PDBu; Sigma) was used at 1 μM for 6 h.

**cDNA constructs, RNA interference and transfection experiments**

GFP-tagged forms of LIMK1, LIMK2 and Talin have been previously described (Worth et al., 2010; Jayo et al, 2012). Lifeact–Ruby/GFP in a lentiviral vector were used and virus generated as described (Scales et al, 2013). Wild-type human podoplanin subcloned into pEGFP-N1 vector has been described elsewhere (Martin-Villar et al, 2006). Lentiviral vectors containing wild-type podoplanin and mutant constructs fused to GFP were generated by PCR amplification using primers that carry convenient restriction sites to facilitate subcloning into the pLNT-SFFV vector (Demaison et al, 2002). Human full-length wild-type podoplanin was cloned into the pLNT-SFFV vector using the In-Fusion HD cloning system (Clontech). Oligonucleotides used for amplification of all these constructs are described in Table E2 in the supplemental material. Short-hairpin RNAs (shRNAs) targeting human podoplanin mRNA cloned into pLKO.1 vector were previously described (Martin-Villar et al, 2010) and the target sequences are included in Table E3.

Transfections in HN5 or HEK293T cells (for lentiviral production) were performed using Fugene 6 transfection reagent (Roche) at a 3:1 ratio (μl Fugene:μg DNA). Cells were used for the indicated assays 24-48h after transfection.

Lentiviral supernatants were produced in HEK293T cells and used as described in (Martin-Villar et al, 2010). For human siRNA experiments in HN5 cells, annealed siRNA oligonucleotides specific to human podoplanin, ezrin, moesin, RhoA and RhoC (described in Table E3) were transfected with 100 μM oligofectamine (Invitrogen) in Opti-MEM reduced-serum medium (Invitrogen). Cells were incubated at 37ºC for 6 h before the addition of FBS to 10%. All the assays were conducted at 72 h after transfection.

**Western blotting and pull down assays**

Cells were lysed in 2% SDS buffer containing 50 mM Tris-HCl pH 7.4, 100 mM NaCl, 5 mM MgCl_2_, 5 mM CaCl_2_ and 2% SDS, and supplemented with pretease inhibitor cocktail set I (Calbiochem) and phosphatase inhibitor complex PhosSTOP from Roche. Protein lysate concentration was determined with the BCA assay (Pierce, Rockford, IL, USA). Samples containing the same amount of protein (10–30 μg) were run on 10% or 12,5% SDS-PAGE and transferred to Immobilon P membranes (Millipore). Membranes were blocked in Tris-buffered saline and Tween 20 solution (TBS-T) and 5% non fat milk or 3% BSA and incubated with specified primary antibodies overnight at 4ºC. The immunoblots were washed in TBS-T and incubated for 1 h with horseradish peroxidase-conjugated secondary antibody at room temperature. Membranes were then washed with TBS-T and visualised with ECL substrate reagent (Pierce).

The level of active RhoA (RhoA-GTP) and RhoC (RhoC-GTP) in cell lysates was measured using a GST fusion protein with the Rho-binding domain of Rhotekin (GST-C21). For Rac1 and Cdc42 activities, a GST fusion protein of the binding domain of PAK (GSTPAK) was used. Assays were performed as described by Sander and co-workers (Sander et al, 1998).

**REFERENCES**

Burger KL, Davis AL, Isom S, Mishra N, Seals DF (2011) The podosome marker protein Tks5 regulates macrophage invasive behavior. *Cytoskeleton (Hoboken)* **68:** 694-711

Demaison C, Parsley K, Brouns G, Scherr M, Battmer K, Kinnon C, Grez M, Thrasher AJ (2002) High-level transduction and gene expression in hematopoietic repopulating cells using a human immunodeficiency [correction of imunodeficiency] virus type 1-based lentiviral vector containing an internal spleen focus forming virus promoter. *Human gene therapy* **13:** 803-813

Jayo A, Parsons M, Adams JC (2012) A novel Rho-dependent pathway that drives interaction of fascin-1 with p-Lin-11/Isl-1/Mec-3 kinase (LIMK) 1/2 to promote fascin-1/actin binding and filopodia stability. *BMC biology* **10:** 72

Sander EE, van Delft S, ten Klooster JP, Reid T, van der Kammen RA, Michiels F, Collard JG (1998) Matrix-dependent Tiam1/Rac signaling in epithelial cells promotes either cell-cell adhesion or cell migration and is regulated by phosphatidylinositol 3-kinase. *The Journal of cell biology* **143:** 1385-1398

Scales TM, Jayo A, Obara B, Holt MR, Hotchin NA, Berditchevski F, Parsons M (2013) alpha3beta1 integrins regulate CD151 complex assembly and membrane dynamics in carcinoma cells within 3D environments. *Oncogene* **32:** 3965-3979

Worth DC, Hodivala-Dilke K, Robinson SD, King SJ, Morton PE, Gertler FB et al. Alpha v beta3 integrin spatially regulates VASP and RIAM to control adhesion dynamics and migration. The Journal of cell biology 2010; 189: 369-383.

**S**UPPLEMENTAL TABLES

| **Table E1.-** Characteristics of SCC cell lines | | | |
| --- | --- | --- | --- |
| **Cell line** | **Origin** | **Culture conditions** | **Reference** |
| HaCaT | Immortalised keratinocytes from adult skin | 10% FBS DMEM | Boukamp, et al., 1988 |
| HN30 | Pharynx | 10% FBS DMEM | Cardinali et al., 1995 |
| HN19 | Lymph Node metastasis | 10% FBS DMEM | Cardinali et al., 1995 |
| SCC9 | Tongue | 10%FBS KGF | Rheinwald et al., 1981 |
| SCC13* | Facial epidermis | Keratinocyte-SFM^1^ and 10%FBS KGF^2^ | Rheinwald et al., 1981 |
| A253 | Submandibular | 10% FBS DMEM | ATCC ref. HTB-41 |
| Fadu | Pharynx | 10% FBS DMEM | ATCC ref. HTB-43 |
| HN5 | Tongue | 10% FBS DMEM | Easty et al. 1981 |
| MDA-MB-231 | Breast adenocarcinoma cel | 10% FBS DMEM | ATCC ref. HTB-26 |

^1^Keratinocyte SFM: Life Technologies Cat No. 17005-075.

^2^KGF: DMEM/F12 (3:1) supplemented with 10% FBS, 2mM L-glutamine, 1% Pen-Strep, 1.36 ng/ml triiodo-L-thyronine, 5μg/ml Insulin, 0,5 μg/ml hydrocortisone, 18 nM Adenine and 10ng/ml EGF.

* SCC13 cells were maintained in Keratinocyte-SFM. For invadopodia experiments cells were grown in KGF medium.

­­

| **Table E2**.- Oligonucleotides used for constructs | | | | |
| --- | --- | --- | --- | --- |
| **Constructs** | **Template** | **Oligonucleotide** | **Restriction sites** | **Final plasmids** |
| P_WT_-GFPlv  P_ΔCT_-GFPlv  P_QN.N_-GFPlv  P_G137L_-GFPlv | pEGFP-P_WT_  pEGFP-P_ΔCT_  pEGFP-P_QN.N_  pEGFP-P_G137L_ | 5´GATTATGATC**ACGCGT**CGCGG 3´  5´CTACCGGACTC**AGATCT**CGAGCTCA 3´ | MluI  BglII | pLNT P_WT_  pLNT P_ΔCT_  pLNT P_QN.N_  pLNT P_G137L_ |
| P_ΔEc_-GFPlv | pEGFP-P_ΔEc_ | 5´TGAACCGTC**GGATCC**GCTAGCATGTG 3´  5´CCGGGCC**ACGCGT**ACCTTAGGGC 3´ | BamHI  MluI | pLNT P_ΔEc_ |
| hPDPN | pcDNA3-hPDPN | 5´TCGCGGCCGCACGCGTCTCGAGGGCCACAGAAGTCAGAA 3´  5´GCCTGGTACC**ACGCGT**GATCCACTAGTAACGGCCGCCAG 3` | MluI | pLNT hPDPN |

**Table E3.-** siRNAs and shRNAs sequences

| **Name and specificity** | **siRNA oligonucleotides** |
| --- | --- |
| Podoplanin siRNA 1 | 5’ GAAAGACCGUUCACCAGAC 3’ |
| Podoplanin siRNA 2 | 5’ CUAACACUGGACCAUUGGA 3’ |
| Ezrin siRNA 1 | 5´CAAGAAGGCACCUGACUUUUU 3´ |
| Ezrin siRNA 2 | 5´GAUCAGGUGGUAAAGACUAUU 3´ |
| Moesin siRNA 1 | 5´AUAAGGAAGUGCAUAAGUCUU 3´ |
| Moesin siRNA 2 | 5´UCGCAAGCCUGAUACCAUUUU 3´ |
| RhoA siRNA 1 | 5´AUGGAAAGCAGGUAGAGUU 3´ |
| RhoA siRNA 2 | 5´GAACUAUGUGGCAGAUAUC 3´ |
| RhoA siRNA 3 | 5´GAGAUAUGGCAAACAGGAU3´ |
| RhoC siRNA 1 | 5´AUAAGAAGGACCUGAGGCAUU 3´ |
| RhoC siRNA 2 | 5´GGAUCAGUGCCUUUGGCUAUU 3´ |
| RhoC siRNA 3 | 5´GAGAGCUGGCCAAGAUGAAUU 3´ |
| Podoplanin shRNA 1 | 5´AATAACCGAAGGAAAGACCGT 3´ |
| Podoplanin shRNA 2 | 5´AAGCAGCTTGACTAAATACCT 3´ |
| Podoplanin shRNA 3 | 5´AAGACCGTTCACCAGACTTGG 3´ |
| Podoplanin shRNA 4 | 5´AACACTGGACCATTGGATCGA 3´ |

**SUPPLEMENTAL FIGURE LEGENDS**

**Figure S1.-** **Podoplanin localises at invadopodia adhesion rings in SCC cells. (A)** Confocal images showing specific localization of endogenous podoplanin in HN5 cells. Bars = 5 μm. Confocal images showing vinculin **(B)** or GFP-Talin **(C)** adhesion rings surrounding the invadopodia actin core in HN5 cells. Bars = 10 μm and 4 μm (right panels). **(D)** The human monocytic THP-1 cell line was used to investigate the presence of podoplanin at podosomes. These cells differentiate to macrophages and form podosomes upon stimulation with PDBu (Phorbol 12,13-dibutyrate). The panel shows podoplanin expression by Western blot in THP-1 cells. Endogenous podoplanin was not detected in undifferentiated or differentiated THP-1 cells after stimulation. In the absence of PDBu (0h) the majority of THP-1 cells are in suspension (S; undifferentiated status) while very few cells are attached (A) to the substratum. PDBu-induced differentiation (24-72h) results in cell attachment and no cells are then found in the culture medium. Note that GAPDH levels were used as readout of the changes in cell attachment during PDBu-induced differentiation, and are not used as loading control. **(E)** To further confirm the apparent absence of podoplanin at podosomes of differentiated THP-1 cells, ectopic expression of PDPN-GFP was achieved by lentiviral infection. THP-1 cells expressing PDPN-GFP were differentiated by treatment with 1 μM PDBu for 6 hours and left to adhere to the coverslips overnight. Confocal images show that, although PDPN-GFP was concentrated at actin-rich cell membrane projections of differentiated THP-1 cells, no podoplanin specific localization to podosome adhesion rings or to the actin core was observed. Bars = 5 μm and 1 μm (right panels).Graphs in A-C and E indicate fluorescent intensity (in arbitrary units) of each marker over the indicated line scan. Data shown are representative from 5-7 invadopodia analysed per condition from two independent experiments.

**Figure S2.- Podoplanin is required for efficient invadopodium-associated matrix degradation in SCC13 cells. (A)** Localization of podoplanin fused to GFP at invadopodia of SCC13 cells. Note podoplanin rings associated to active invadopodia (lower panels). Graph indicates fluorescent intensity (in arbitrary units) of each marker over the indicated line scan. Data shown are representative from 5-7 invadopodia analysed per condition from two independent experiments. **(B)** Expression levels of podoplanin in SCC13 podoplanin knockdown and over-expressing cells by Western blot. **(C)** Gelatin degradation assay (6h) showing the reduced ability of SCC13-PDPNsh cells to degrade the matrix compared to control cells, and enhanced invadopodia-mediated matrix degradation after podoplanin over-expression in SCC13-PDPN cells. Bar = 50 μm. **(D-E)** Graphs showing the quantification of the gelatin degradation assay depicted in C. The results shown are the means ± SEM of n ≥ 100 cells (left panels in D-E) or n ≥ 100 invadopodia (middle and right panels in D-E) for each condition over three independent experiments. *p<0.01; **p<0.001; ***p<0.0001.

**Figure S3.- Podoplanin enhances invadopodium-associated matrix degradation in MDA-MB-231 cells. (A)** Podoplanin expression by Western blot in MDA-MB-231 parental cells and podoplanin or podoplanin-GFP cell transfectants. Note that endogenous podoplanin is absent in MDA-MB-231 cells. **(B-C)** Confocal images showing podoplanin-GFP specific localization at invadopodial rings of MDA-MB-231 cells. **(D)** Graphs representing the fluorescent intensity (in arbitrary units) of each marker over the indicated line scan. Data shown are representative from 5-7 invadopodia analysed per condition from two independent experiments. **(E)** Gelatin degradation assay (4h) showing enhanced invadopodia-mediated matrix degradation after podoplanin overexpression in 231-PDPN cells. Bar = 10 μm. **(F)** Graphs showing the quantification of the gelatin degradation assay depicted in E. The results shown are the means ± SEM of n ≥ 100 cells (top panel) or n ≥ 100 invadopodia (bottom panel) for each condition over three independent experiments. *p<0.01; **p<0.001; ***p<0.0001.

**Figure S4.- Localization of podoplanin at invadopodia of control or PDPN-GFP HaCaT cell transfectants. (A-B)** Confocal images showing invadopodia of control GFP and PDPN-GFP expressing HaCaT cells. The specific localization of PDPN-GFP at invadopodia of HaCaT cells is also shown. Note podoplanin rings associated to active invadopodia (lower panels in A) or surrounding the actin/cortactin core (lower panels in B). Bars = 10 and 2 μm. Graphs indicate fluorescent intensity (in arbitrary units) of each marker over the indicated line scan. Data shown are representative from 5-7 invadopodia analysed per condition from two independent experiments.

**Figure S5.- Invadopodia reassembly rates in podoplanin knock-down cells after serum starvation.** Cells were grown on crosslinked unlabelled gelatin overnight upon serum starvation. Invadopodia reassembly was then stimulated after washout with normal medium containing 10% FBS. The graph shows the quantification of the number of cells with invadopodia at the indicated time points. The results shown are the means ± SEM of n ≥100 cells for each condition and time point of one representative experiment.

**Figure S6.- Localization of podoplanin and ezrin/moesin in invadopodia of ezrin/moesin or podoplanin knockdown cells. (A)** Confocal images showing PDPN-GFP localization in invadopodia of ezrin and moesin knock-down cells. Bars=10 μm (upper panels) and 5 μm (lower panel). **(B)** Confocal images showing ezrin localization in invadopodia of podoplanin-depleted cells. Bars=10 μm (upper panels) and 2 μm (lower panel). Graphs in A-B indicate fluorescent intensity (in arbitrary units) of each marker over the indicated line scan. Data shown are representative from 5-8 invadopodia analysed per condition from two independent experiments.

**Figure S7.- Podoplanin regulates cofilin phosphorylation at Ser3 through the RhoC/ROCK/LIMK pathway. (A)** Rac1 and Cdc42 bound to GTP affinity pull-down assays. **(B)** Quantification of RhoA- and RhoC-GTP expression levels from the blots depicted in Figure 9A relative to total levels and GAPDH loading control was performed by densitometric analysis. Statistically significant differences were determined by one-way analysis of variance (ANOVA). p<0,0001 for RhoA and p<0,001 for RhoC. **(C)** Quantification of RhoA and RhoC expression levels from the blots depicted in Figure 9E. Statistically significant differences were determined by ANOVA. p<0,0001. **(D)** Determination of pCofilinS3 levels during the stages of invadopodia formation in control and podoplanin-depleted HN5 cells. Complementary quantitative analysis is shown in Fig 9D. **(E)** Effects of RhoA and RhoC knock-down in invadopodia-mediated degradation of HN5 cells. These images complement the quantitative analysis depicted in Fig 9G). **(F)** Downregulation of RhoA in HN5 cells leads to increased Rac1 and Cdc42 expression. Western blot analysis of Rac1 and Cdc42 in RhoA and RhoC depleted cells.

**Figure S8.- Control of invadopodia stability by podoplanin-dependent regulation of cofilin activity.** Early recruitment of podoplanin to the invadopodium is mediated by lipid rafts following the initial appearance of actin puncta. Podoplanin accumulation into the invadopodium promotes the recruitment of ERM proteins, which in turn mediate the assembly of podoplanin rings and its stabilisation around the actin core. Activation RhoC is then induced by podoplanin, leading to cofilin phosphorylation through ROCK/LIMK. Cofilin inactivation by phosphorylation at Ser3 prevents the excessive severing and actin depolymerisation allowing the stabilisation of the invadopodium structure and its subsequent maturation.

**SUPPLEMENTAL MOVIES**

**Fig4videoS1**.- **Podoplanin dynamics at invadopodia.** HN5 cells expressing PDPN-GFP (green) and Lifeact-Ruby (red) were plated on crosslinked gelatin and analysed by confocal microscopy (NIKON A1; Plan Apo VC x60 Oil 1.4NA objective). Images were taken every 30 s for 3 h. Kymographs of the indicated invadopodia (1, 2 and 3) are shown in Figure 4B. Playback rate presented is 10 frames/s.

**Fig4videoS2**.- **Podoplanin dynamics during matrix degradation.** HN5 cells expressing PDPN-GFP (green) were plated on TRITC crosslinked gelatin. The basal cell surface in contact with the gelatin was analysed by confocal microscopy (NIKON A1; Plan Apo VC x60 Oil 1.4NA objective). Images were taken every 30 s for 3 h. Kymographs of the indicated invadopodia (1, and 2) are shown in Figure 4D. Playback rate presented is 10 frames/s.

**Fig5videoS3.- Invadopodia dynamics of Control and podooplanin-depleted cells.-** Control Sc and PDPNsh HN5 cells expressing Lifeact-GFP were seeded on crosslinked gelatin and analysed by confocal microscopy (NIKON A1; Plan Apo VC x60 Oil 1.4NA objective). Images were taken as one image per 30 s for 3 h, and are presented at playback rates of 10 frames/s.
