# Supplementary figures and images for: Podoplanin mediates ECM degradation by squamous carcinoma cells through control of invadopodia stability
Source: Oncogene. 2014 Dec 8;34(34):4531–44. doi: 10.1038/onc.2014.388 (PMC4430312; doi:10.1038/onc.2014.388)

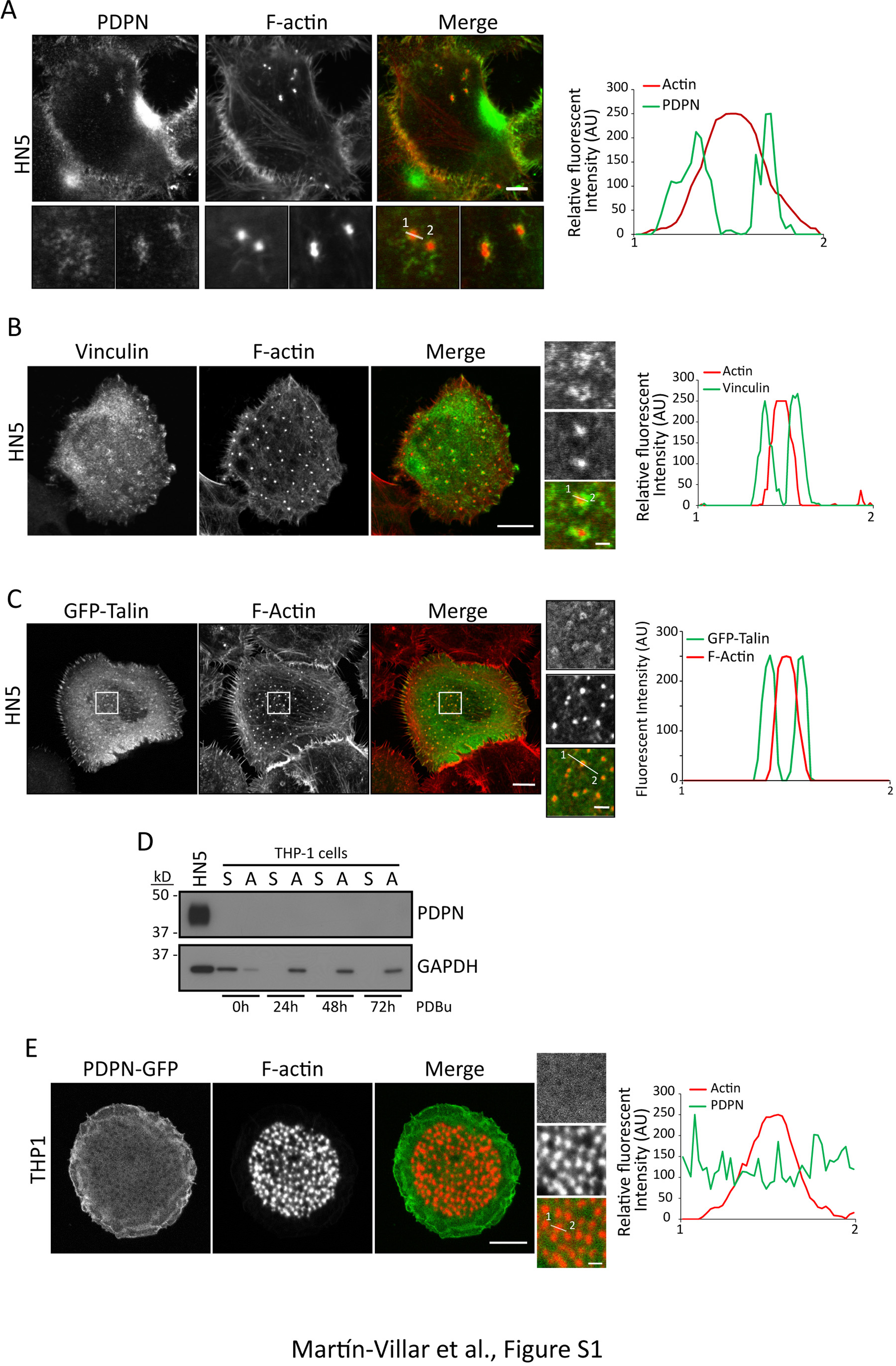

Supplement: Supplementary Figure S1 [file onc2014388x2.tif]

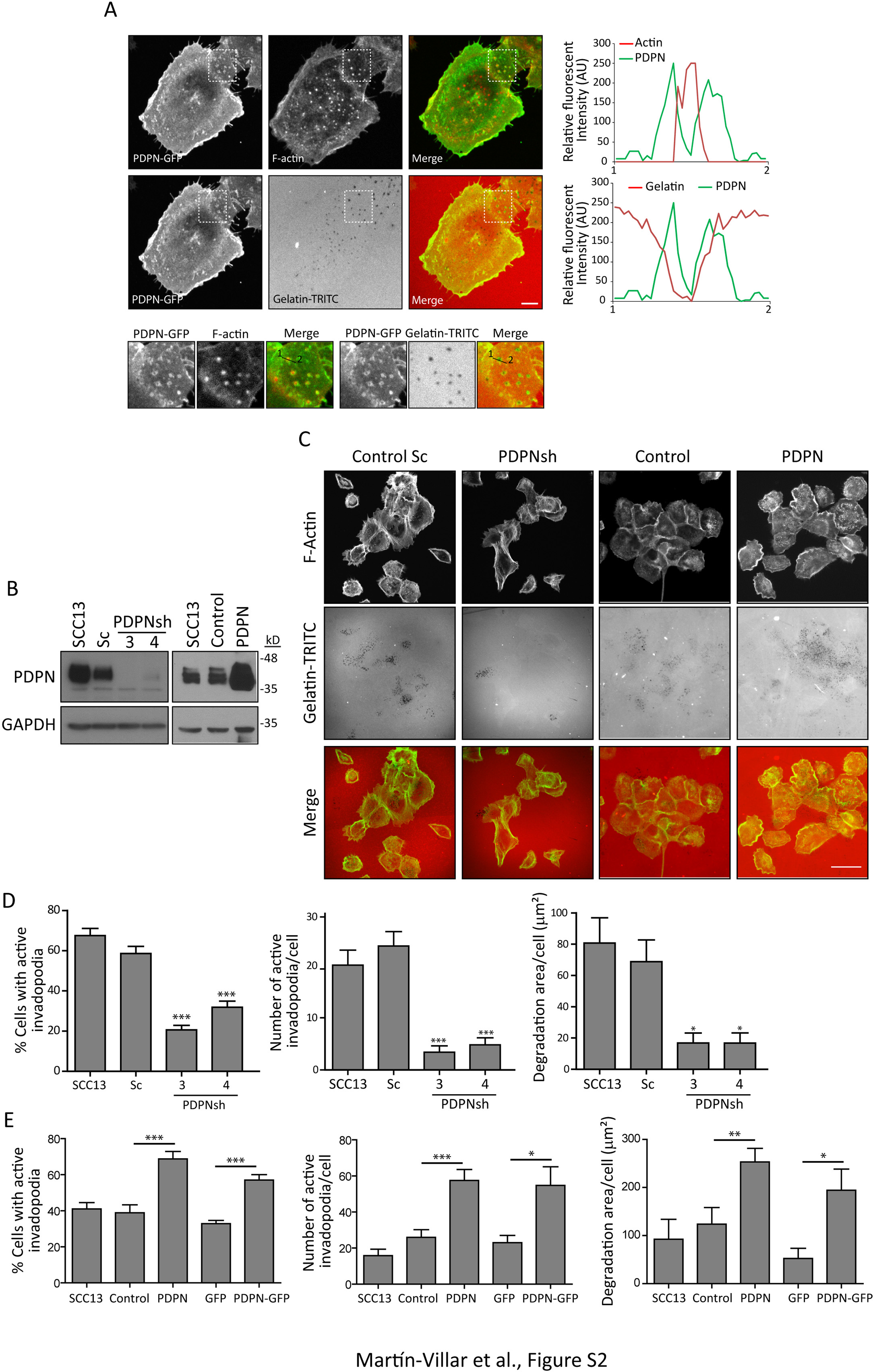

Supplement: Supplementary Figure S2 [file onc2014388x3.tif]

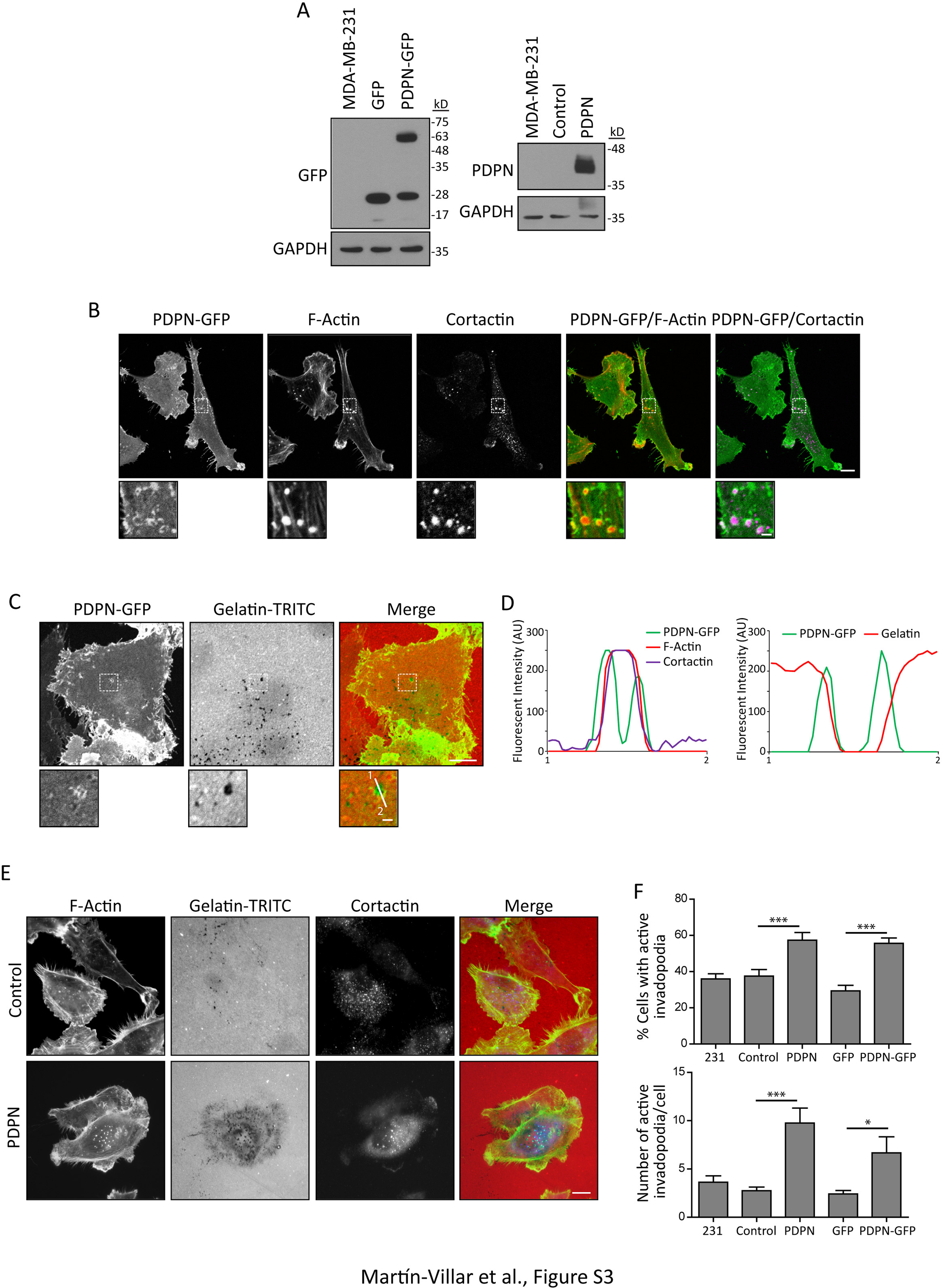

Supplement: Supplementary Figure S3 [file onc2014388x4.tif]

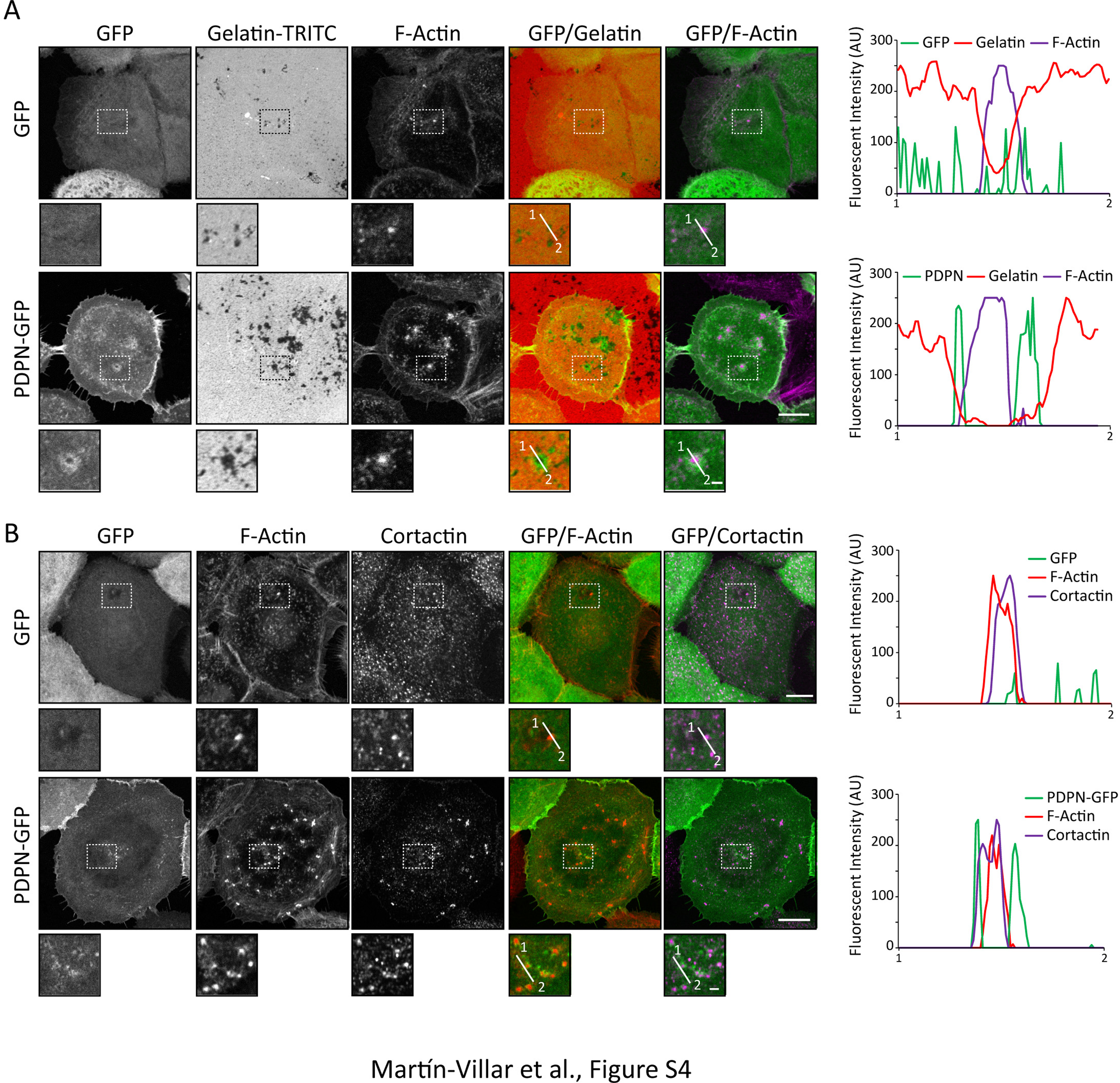

Supplement: Supplementary Figure S4 [file onc2014388x5.tif]

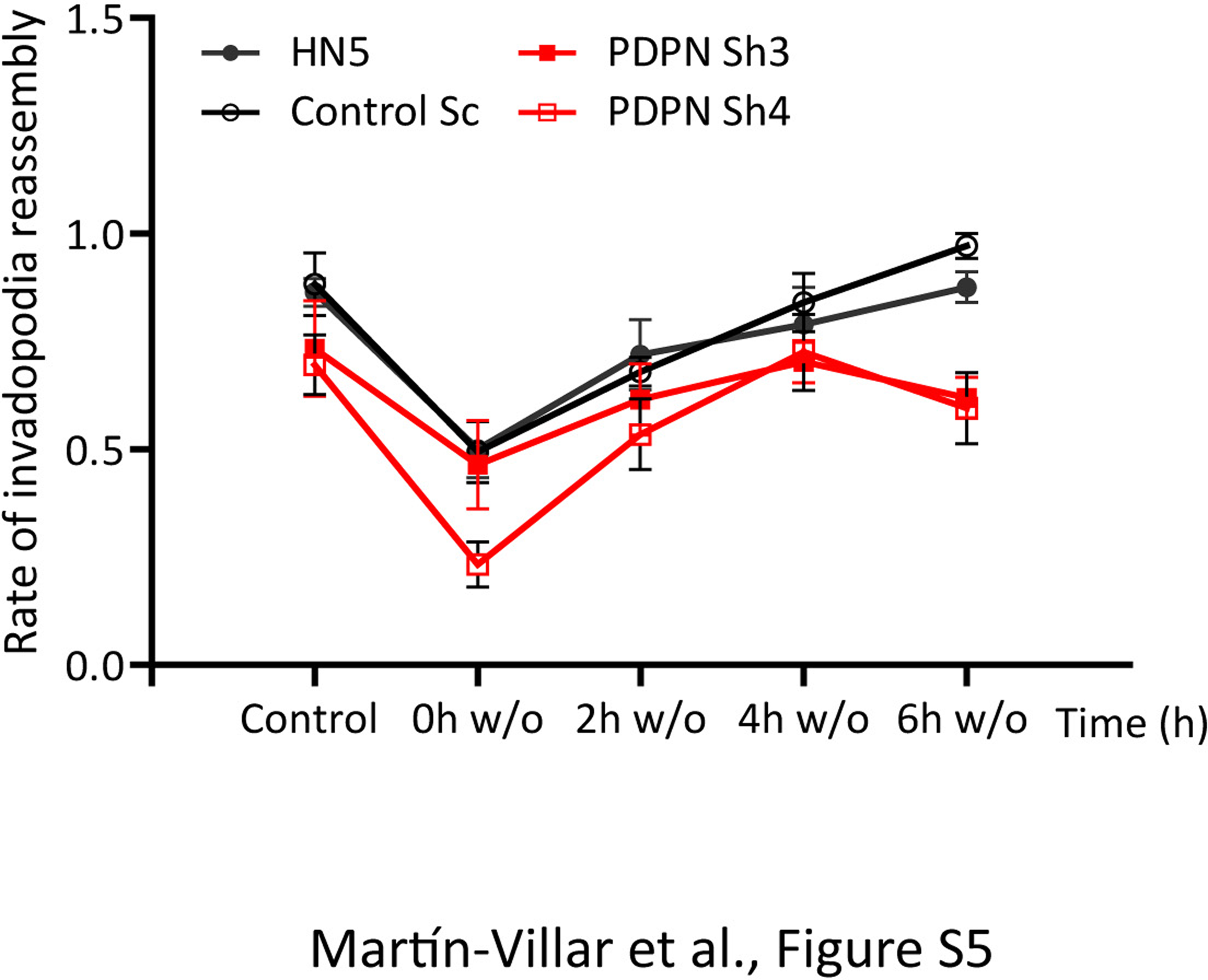

Supplement: Supplementary Figure S5 [file onc2014388x6.tif]

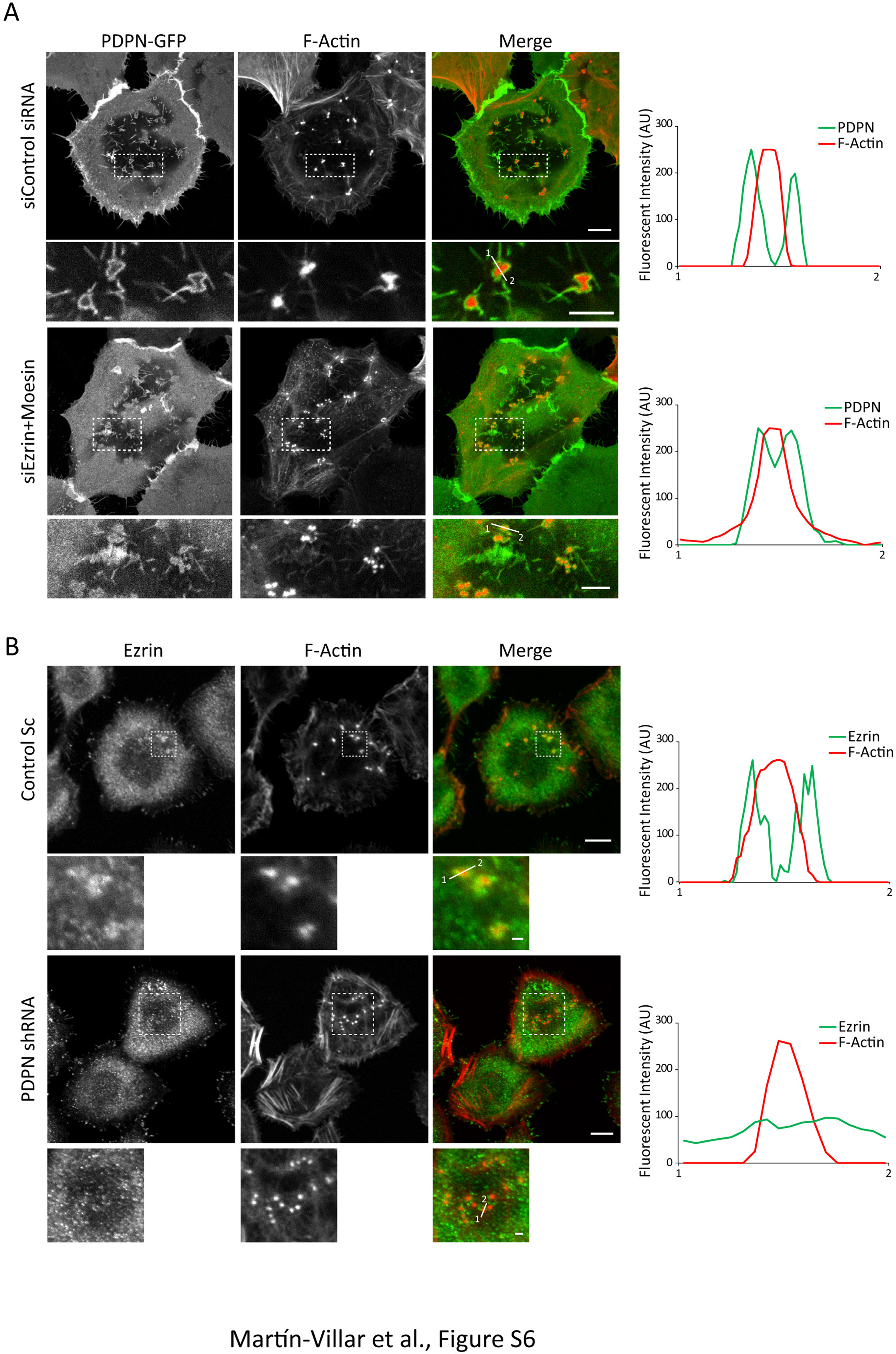

Supplement: Supplementary Figure S6 [file onc2014388x7.tif]

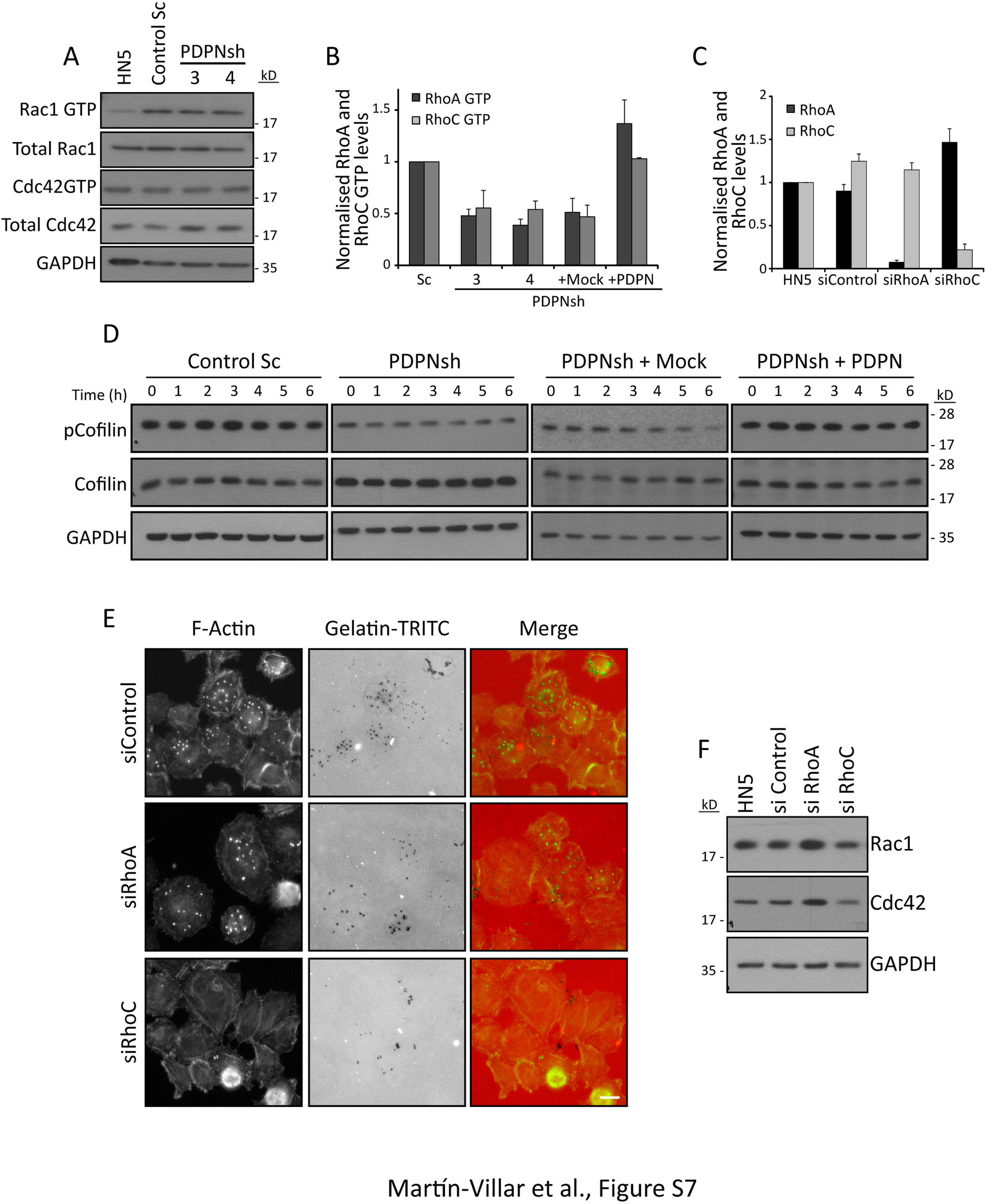

Supplement: Supplementary Figure S7 [file onc2014388x8.tif]

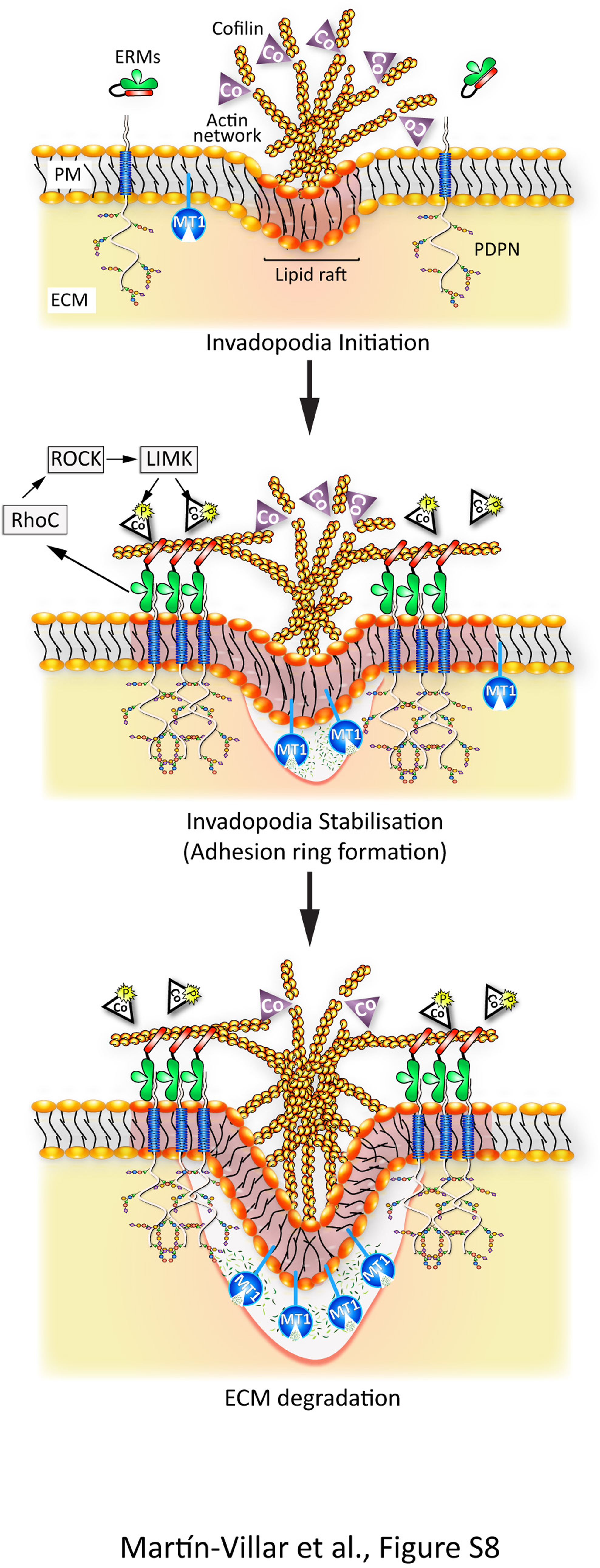

Supplement: Supplementary Figure S8 [file onc2014388x9.tif]
